# Transgene Induced Co-Suppression during Vegetative Growth in *Cryptococcus neoformans*

**DOI:** 10.1371/journal.pgen.1002885

**Published:** 2012-08-16

**Authors:** Xuying Wang, Ping Wang, Sheng Sun, Sabrina Darwiche, Alexander Idnurm, Joseph Heitman

**Affiliations:** 1 Department of Molecular Genetics and Microbiology, Duke University Medical Center, Durham, North Carolina, United States of America; 2 The Research Institute for Children, Children's Hospital, New Orleans, Louisiana, United States of America; 3 Department of Pediatrics and Department of Microbiology, Immunology, and Parasitology, Louisiana State University Health Sciences Center, New Orleans, Louisiana, United States of America; 4 Division of Cell Biology and Biophysics, School of Biological Sciences, University of Missouri–Kansas City, Kansas City, Missouri, United States of America; The University of North Carolina at Chapel Hill, United States of America

## Abstract

Introduction of DNA sequences into the genome often results in homology-dependent gene silencing in organisms as diverse as plants, fungi, flies, nematodes, and mammals. We previously showed in *Cryptococcus neoformans* that a repeat transgene array can induce gene silencing at a high frequency during mating (∼50%), but at a much lower frequency during vegetative growth (∼0.2%). Here we report a robust asexual co-suppression phenomenon triggered by the introduction of a *cpa1::ADE2* transgene. Multiple copies of the *cpa1::ADE2* transgene were ectopically integrated into the genome, leading to silencing of the endogenous *CPA1* and *CPA2* genes encoding the cyclosporine A target protein cyclophilin A. Given that *CPA1*-derived antisense siRNAs were detected in the silenced isolates, and that RNAi components (Rdp1, Ago1, and Dcr2) are required for silencing, we hypothesize that an RNAi pathway is involved, in which siRNAs function as *trans* factors to silence both the *CPA1* and the *CPA2* genes. The silencing efficiency of the *CPA1* and *CPA2* genes is correlated with the transgene copy number and reached ∼90% in the presence of >25 copies of the transgene. We term this transgene silencing phenomenon asexual co-suppression to distinguish it from the related sex-induced silencing (SIS) process. We further show that replication protein A (RPA), a single-stranded DNA binding complex, is required for transgene silencing, suggesting that RPA might play a similar role in aberrant RNA production as observed for quelling in *Neurospora crassa*. Interestingly, we also observed that silencing of the *ADE2* gene occurred at a much lower frequency than the *CPA1/2* genes even though it is present in the same transgene array, suggesting that factors in addition to copy number influence silencing. Taken together, our results illustrate that a transgene induced co-suppression process operates during *C. neoformans* vegetative growth that shares mechanistic features with quelling.

## Author Summary

The development of gene transfer methods allows the production of transgenic lines in myriad eukaryotes. Frequently, transgenic DNA is integrated into the genome and transmitted as a heritable Mendelian trait. However, the introduced transgenes are in some cases not expressed (silenced). In addition, transgenes can also provoke silencing of endogenous genes with which they share sequence homology. This phenomenon was first observed in plants and named co-suppression. In fungi the best-documented co-suppression phenomenon occurs in vegetative tissue of the filamentous fungus *Neurospora crassa* and is termed quelling. Here we report a robust asexual co-suppression pathway that operates in the pathogenic fungus *Cryptococcus neoformans* and shares molecular components with quelling. Compared with the sex induced silencing (SIS) phenomenon previously discovered in *C. neoformans*, which efficiently silences genes during mating (∼50%) but not during vegetative growth (∼0.2%), asexual co-suppression operates efficiently during vegetative growth to suppress transgene expression and may also silence transposons and other repetitive sequences.

## Introduction

In genetically modified plants and fungi, introduced transgenes are in some cases silenced and can also cause silencing of endogenous genes if they share sufficient homology [1]. This phenomenon has been termed co-suppression or homology-dependent gene silencing (HDGS), and usually occurs when multiple copies of particular sequences are present in the genome [2], [3]. Depending on the mechanistic level at which silencing occurs, HDGS has been distinguished into two types: transcriptional gene silencing and post-transcriptional gene silencing (TGS and PTGS, respectively) [1]. During TGS, repression of transcriptional initiation is normally achieved by establishing an epigenetic inactivation state characterized by altered methylation patterns or chromatin structure. In contrast to TGS, genes silenced by PTGS are normally transcribed but their transcripts do not accumulate, as a consequence of rapid degradation. An RNAi degradation process is central to PTGS, in which a dsRNA intermediate homologous to the target gene or transgene is generated and processed into siRNAs of 21–25 nucleotides. siRNAs subsequently guide gene silencing in a sequence-specific manner [4], [5].

In fungi, a co-suppression like phenomenon referred to as quelling [6], [7] occurs in *Neurospora crassa* vegetative tissue, while other related silencing phenomena, repeat-induced point mutation (RIP) [8] and methylation induced pre-meiotically (MIP) [9], [10] are active in the premeiotic phase during the sexual cycles of *N. crassa*, *Ascobolus immersus*, and *Coprinopsis cinerea*, respectively. Quelling, RIP, and MIP are well-documented cases of HDGS showing variability in developmental timing, and are also mechanistically heterogeneous. MIP in *A. immersus* and *C. cinerea* and RIP in *N. crassa* involve a similar molecular mechanism to inactivate repetitive sequences, except that repeat sequences are methylated in MIP [11], whereas RIP involves inactivating the methylated sequences by introduction of C-to-T (G-to-A) transition mutations [12]. Quelling is an RNAi-related PTGS process that is induced by siRNAs and requires the core RNAi components, including Argonaute, Dicer-like proteins, and RNA-dependent RNA polymerase (RdRP) [5], [13], [14], [15]. In quelling, the silenced loci can act in *trans*, leading to silencing of all homologous genes.

Co-suppression phenomena similar to quelling have been widely described in *Arabidopsis*, *Drosophila*, and *C. elegans* [16], [17]. In many cases, these phenomena are induced by either highly expressed single copy transgenes or highly repetitive transgene arrays present in the genome, and gene silencing is often linked to an RNAi pathway [13], [18], [19]. In addition to regulating transgene expression, co-suppression is also considered a host genome defense mechanism acting against invading parasitic selfish DNA, such as transposons and viruses. For example, Piwi-interacting RNAs (piRNAs) that bind to the Piwi proteins of the Argonaute superfamily are required for silencing transposons in the animal germline via an RNAi-PTGS process [20], [21]. Additionally, viruses carrying homology to nuclear sequences can trigger PTGS in plants, resulting in silencing of both viral genes and endogenous genes [22], [23].

Transgene-induced gene silencing has also been observed in *Cryptococcus neoformans*. In a previous study, we reported the discovery of sex induced silencing (SIS) in *C. neoformans*, a novel silencing mechanism triggered by a tandem multi-copy insertion of a *SXI2* **a**-*URA5* transgene [24]. SIS is highly active during the sexual cycle and requires the central components of the RNAi pathway to silence target genes post-transcriptionally [24]. The frequency of SIS is increased with higher transgene copy number, but becomes saturated at ∼50% when more than three copies of a transgene are integrated into the genome. In addition to inactivating the transgene arrays, SIS also functions to squelch transposon activity during the sexual cycle, which is reflected in the observation that an increased transposition/mutation rate was detected in RNAi mutant progeny [24]. In summary, the SIS RNAi pathway has been proposed as a meiotic mechanism to guard genome integrity.

Here we report a robust transgene-induced silencing phenomenon homologous to SIS, but occurring during *C. neoformans* vegetative (asexual) growth. This silencing is more related to co-suppression in plants and quelling in *N. crassa* because no sexual cycle is involved. To distinguish this process from SIS, we named this silencing phenomenon asexual co-suppression. *Cryptococcus* co-suppression is also initiated by the integration of a transgene array into the genome, and inactivates genes that are homologous to DNA sequences introduced by the transgene. We observed that the silencing efficiency reached ∼90% in the case of more than 25 copies of a transgene. The fact that the suite of RNAi machinery components and the RPA complex are necessary for asexual co-suppression indicates that asexual co-suppression may share a similar molecular mechanism with quelling in *N. crassa*. Notably, when the introduced transgene array contains regions homologous to different target genes, the silencing rates may vary and are gene-specific. These findings provide evidence that a quelling-like asexual co-suppression pathway operates in *C. neoformans*, and occurs at different efficiencies with regard to different target genes. Similar to SIS, asexual co-suppression could be a major mechanism involved in silencing transposons and repetitive sequences in *C. neoforman*s during vegetative growth.

## Results

### Silencing of the cyclophilin A *CPA1* and *CPA2* genes via an ectopic transgene

**Figure 1:**
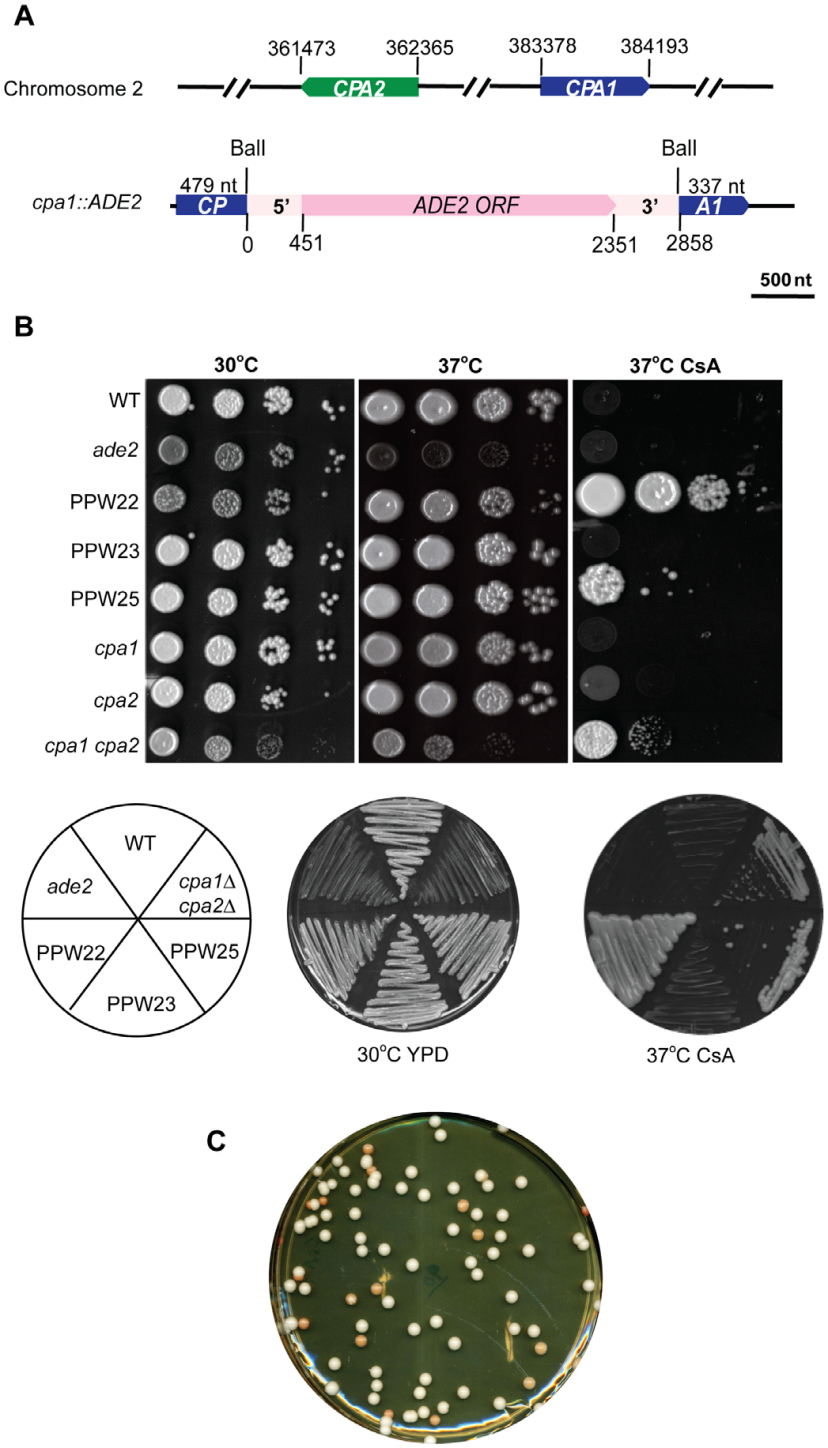
Transgene silencing results in CsA resistance and ade−, red colonies. A) Schematic illustrations of the *CPA1* and *CPA2* genes (upper) and the disruption allele (lower) used in this study. The *CPA1* and *CPA2* genes lie on Chromosome 2 and are 21.01 kb apart and divergently transcribed. An ∼2.9 kb fragment spanning the *C. neoformans ADE2* gene, including the 5′ UTR region (451 nt, light pink box, 0–451), *ADE2* ORF (1.9 kb, pink box, 451–2351), and the 3′ UTR region (507 nt, light pink box, 2351–2858) was obtained by PCR and inserted into the unique BalI site located within the *CPA1* gene (blue box, left box is 479 nt and right box is 337 nt). The black lines indicate the 5′ and 3′ UTR regions of the *CPA1* gene (45 nt and 407 nt respectively). The *cpa1::ADE2* allele was amplified by PCR and transformed into the *ade2* train M049. The scale bar is 500 nt. B) The wild-type strain H99, the *ade2* mutant strain M049, *cpa1*, *cpa2*, and *cpa1 cpa2* mutant strains together with three transformed strains in which the *cpa1::ADE2* disruption allele was integrated ectopically (strains PPW22, PPW23, and PPW25) were 10-fold serially diluted in YPD medium (started with 5×10^6^ cells/ml), and the cell suspensions were spotted onto YPD medium with or without 100 µg/ml cyclosporine A (CsA) and incubated at 30°C and 37°C for four days and photographed (upper). H99, M049, PPW22, PPW23, PPW25, and *cpa1 cpa2* mutant strains were also grown on YPD medium with or without 100 µg/ml cyclosporine A (CsA) and incubated at 30°C and 37°C for four days and photographed (lower). C) PPW22 is variegated, forming white and red colonies. PPW22 was inoculated into a 5 ml liquid overnight culture and then plated on YPD solid medium. The plate was photographed after 3 days growth at 25°C.

*CPA1* and *CPA2* are two homologous genes encoding two conserved cyclophilin A proteins, Cpa1 and Cpa2, in *C. neoformans*. They are both located on chromosome 2, linked 21.01 kb apart, and share 85% nucleotide identity in the coding regions (Figure 1A). Previously, we disrupted the *CPA1* and *CPA2* genes, individually and in combination, and determined the functions of cyclophilin A in *C. neoformans* [25]. Both the Cpa1 and Cpa2 cyclophilin A proteins are the targets of the immunosuppressive and antifungal natural product cyclosporine A (CsA); either *cpa1* or *cpa2* single mutant strains remain sensitive to CsA at 37°C but *cpa1 cpa2* double mutants are completely resistant to CsA. Thus, both Cpa1 and Cpa2 mediate CsA inhibition of calcineurin and inhibit growth at 37°C. In addition, the *cpa1 cpa2* mutant is sterile in genetic crosses and formed almost no heterokaryotic filaments, basidia, or basidiospores [25].

**Figure 2:**
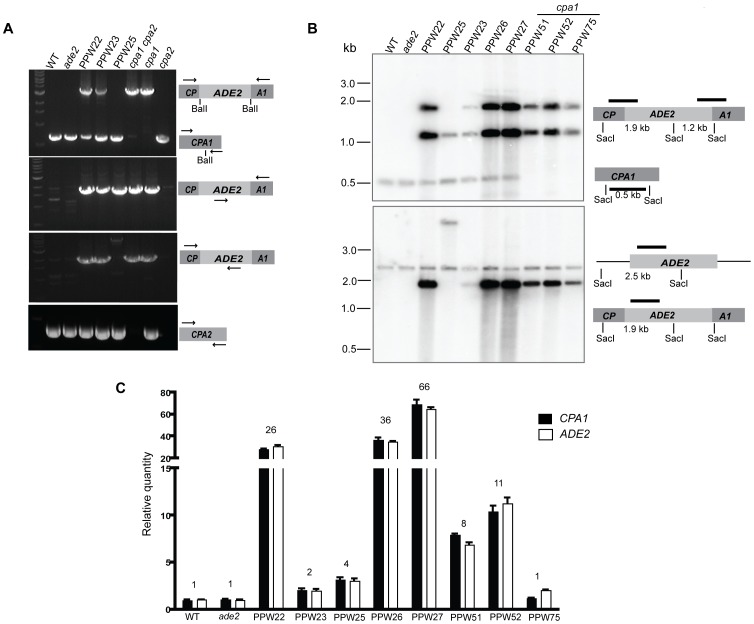
The *cpa1::ADE2* transgenes are ectopically integrated into the genome of the transformed strains in different copy numbers. A) PCR analyses with primers specific to the *CPA1*, *CPA2*, or *cpa1::ADE2* genes were used to demonstrate the presence of the wild-type *CPA1* and *CPA2* genes and the *cpa1::ADE2* transgene in all three transformed strains. The primers employed in PCR analyses are shown as arrows in the right panels. B) DNA from the wild-type, *ade2* mutant, *cpa1* mutant, and eight transformed strains was cleaved with SacI and analyzed by Southern hybridization. When a *CPA1* gene-specific SacI restriction fragment was used as a probe, the wild-type *CPA1* gene hybridized to a 0.5 kb SacI fragment, and 1.25 and 1.9 kb fragments represent the cleavage products of ectopically integrated *cpa1::ADE2* alleles in the transformed strains (upper panel). When hybridized with a probe specific to *ADE2* (lower panel), a 2.5 kb product was detected from the wild-type *ADE2* gene and a 1.9 kb band indicates the cleavage product from the *cpa1::ADE2* transgene. C) Quantitative real-time PCR revealed that different copy numbers of the *cpa1::ADE2* transgene (total numbers of *CPA1* or *ADE2* minus one endogenous copy) are present in the genomes of the PPW22, PPW23, PPW25, PPW26, PPW27, PPW51, PPW52, and PPW75 strains.

Paradoxically, during screens to isolate *cpa1* single mutant strains following introduction of a *cpa1::ADE2* disruption allele (Figure 1A) into the *ade2* strain M049, we observed that ∼25% of Ade+ transformants were resistant to CsA. The *cpa1::ADE2* disruption allele is composed of the *C. neoformans ADE2* gene with 5′ and 3′ UTRs inserted into a full length *cpa1* gene at an internal BalI restriction site (Figure 1A). The Ade+ transformant strain PPW23 remained sensitive to CsA whereas strains PPW22 and PPW25 exhibited various degrees of CsA resistance with PPW22 being the most CsA resistant and viable on YPD medium containing 100 µg/ml CsA at 37°C (Figure 1B). CsA resistance exhibited by some of the transformants was unstable and these colonies were variegated, forming white and red colonies/sectors indicative of repression or loss of the *ADE2* marker gene (Figure 1C). PCR analyses indicated that all of these isolates carried the wild-type length *CPA1* and *CPA2* genes (Figure 2A). Furthermore, we sequenced the *CPA1* and *CPA2* genes from the transformed strains, and found no mutations. Thus, in no case was the CsA resistance phenotype attributable to the disruption or mutation of *CPA1* or *CPA2*. Based on PCR analyses (Figure 2A), the PPW22 and PPW23 isolates contain an intact *cpa1::ADE2* transgene allele, as well as the wild type *CPA1* gene. Isolate PPW25 also carries the transgene, but PCR amplification failed with a primer matched to the 5′ region of the *CPA1* gene, suggesting that mutations or rearrangements affecting this region occurred during integration (Figure 2A). Taken together, we propose that silencing of the endogenous *CPA1* and *CPA2* genes is triggered by introduction of ectopic transgene(s) that share significant homology with both genes.

### A multicopy transgene is required for silencing of the *CPA1* and *CPA2* genes

The Ade+ transformant strains PPW22, PPW23, and PPW25 varied in their ability to grow on YPD medium with CsA at 37°C, even though all contain the *cpa1::ADE2* transgene allele. We explored the hypothesis that the silencing frequency of *CPA1* and *CPA2* is affected by the location/copy number/arrangement of the transgenic alleles present in the genome. In addition to PPW22, PPW23, and PPW25, we analyzed four other transformed strains containing the *cpa1::ADE2* transgene to test our hypothesis. Southern analyses with probes to *CPA1* and *ADE2* were conducted to compare the genomic structures at the *cpa1::ADE2* locus in these transformed strains. As shown in Figure 2B, the endogenous *CPA1* gene hybridized to a *CPA1* specific probe in PPW22, PPW23, PPW25, PPW26, and PPW27 but not in the *cpa1* mutant strains PPW51, PPW52, and PPW75. Furthermore, similar Southern patterns corresponding to the transgene were observed among PPW22, PPW23, PPW26, PPW27 and the *cpa1* mutant. PPW25 exhibited a different transgene hybridization pattern, consistent with the PCR result noted above (Figure 2A), indicating that mutations or gene rearrangement occurred during integration of the *cpa1::ADE2* transgene allele in this isolate. The signals derived from the ectopically integrated *cpa1::ADE2* alleles are 1.25 kb (*ADE2* probe) and 1.9 kb (both *ADE2* and *CPA1* probes), which were more intense in PPW22, PPW26, and PPW27 compared with the signals from the endogenous *CPA1* or *ADE2* genes, indicating that multiple copies of the transgenes have integrated into these genomes (Figure 2B). We further determined the copy number of the *cpa1::ADE2* transgene by quantitative PCR analyses. As shown in Figure 2C, PPW22, the isolate exhibiting a high degree of CsA resistance, carries ∼25 copies of the transgene, while only three copies were found in PPW25 and one copy in PPW23 (Figure 2C). The transgenes in PPW22 are stably inherited during mitotic growth as shown in Figure S1. In addition, PPW26, PPW27, and the *cpa1* mutant strains PPW51 and PPW52 all contain various copy numbers of the transgene: 35 copies in PPW26, 65 copies in PPW27, 7 copies in PPW51, and 10 copies in PPW52, respectively.

**Figure 3:**
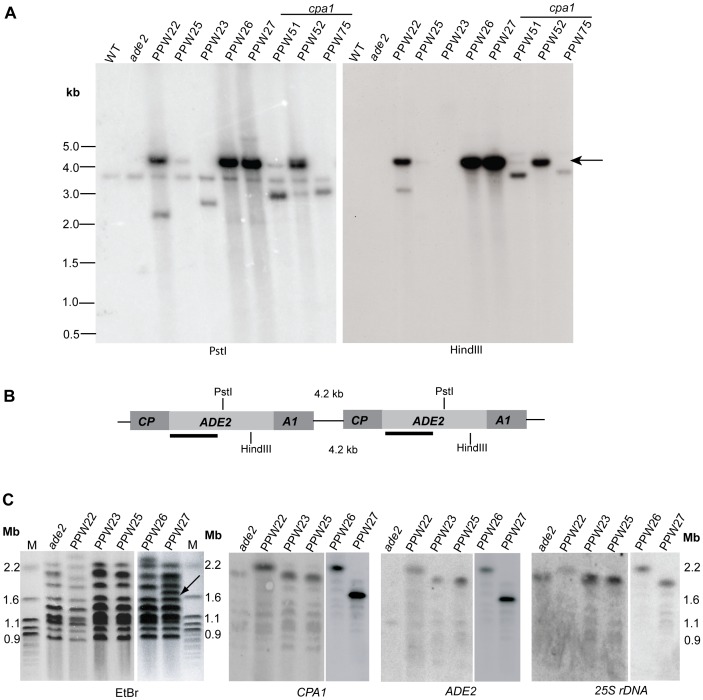
The *cpa1::ADE2* transgenes are arranged as tandem repeats in PPW22, PPW26, and PPW27. A) Digests of genomic DNA from the representative transformed strains. Each DNA sample was cut by PstI (left) and HindIII (right), followed with Southern blot analysis using *ADE2* as a probe. Both PstI and HindIII cleave only once within the *cpa1::ADE2* transgene. An intense ∼4.2 kb band (indicated by arrows) can be detected when the *cpa1::ADE2* transgene is tandemly repeated. B) The deduced arrangement of the tandem repetitive unit of the *cpa1::ADE2* transgene is depicted. The *ADE2* probe used in the top Southern analysis is indicated as black lines. C) Protoplasts from the host untransformed strain (M049) and PPW22, PPW23, PPW25, PPW26, and PPW27 strains were prepared and chromosomal DNA separated by CHEF gel electrophoresis. The ethidium bromide-stained gel is shown in the left panel. To determine the chromosomal locations of the *cpa1::ADE2* locus, the DNA was transferred to a nylon membrane, followed by hybridization with probes to the *CPA1*, *ADE2*, or 25S rDNA genes. The *cpa1::ADE2* transgenes map to chromosome 2 in PPW22, PPW23, PPW25, and PPW26 strains. In PPW27, the *cpa1::ADE2* transgenes are located on a smaller chromosome (indicated by an arrow). M stands for the CHEF DNA size marker.

We also performed Southern analysis with an *ADE2* probe to explore the possible distribution of the transgenes in the genome. As shown in Figure 3A, an ∼4.2 kb band with high intensity was observed in PPW22, PPW26, PPW27, and PPW52, suggesting that the transgenes are likely arranged as tandem repeats in these strains (Figure 3A and 3B). For PPW51, although it carries a 7-copy transgene, it did not yield an intense hybridization band at 4.2 kb, indicating a different transgene arrangement.

To define the location of the transgene, we conducted pulsed-field gel electrophoresis and chromosomal Southern blots with probes directed against the *ADE2* and *CPA1* genes. The chromosomes in the *ade2*, PPW22, PPW23, PPW25, and PPW26 lanes that hybridized to the *CPA1* probe were the same ones that hybridized to the rDNA probe on a duplicate filter, indicating that the transgenes in PPW22, PPW23, PPW25, and PPW26 are located on chromosome 2, on which the rDNA gene cluster and the *CPA1/CPA2* genes reside [26] (Figure 3C). This interpretation is further supported by the finding that hybridization with an *ADE2*-specific probe revealed that the location of the *cpa1::ADE2* transgene in the four transformants is on chromosome 2. PPW27 is distinct from other transformed strains in that the transgenes are located on a chromosome smaller than chromosome 2.

We then explored the correlation between the silencing efficiency and the transgene copy number by measuring spontaneous CsA resistance after strains bearing different copies of the transgene were grown on rich (YPD) medium. With ∼65 copies of the transgene, PPW27 exhibited the highest silencing rate (∼95%). PPW22 (∼25 copies) and PPW26 (∼35 copies) showed a 90% silencing frequency, whereas the frequency was reduced to ∼0.1% in PPW25, in which three transgenes were present. No CsA resistant colonies were observed from PPW23, which contains a single transgene (Table 1). Interestingly, in the *cpa1* mutant strains PPW51 and PPW52 that contain similar copy numbers of the transgene (7 vs. 10), they exhibited very different silencing efficiency: 0.002% in PPW51 and 1% in PPW52. Taken together, our results showed a correlation between the copy number of the transgene and the intensity of co-suppression; however, this correlation may not be strict, as indicated by the low silencing rate in PPW51. In this strain, other factors such as the arrangement of the transgene may also affect silencing frequency.

**Table 1:** The copy number of the *cpa1::ADE2* transgene is correlated with the silencing frequency of *CPA1/CPA2*.

	PPW23	PPW25	PPW51	PPW52	PPW22	PPW26	PPW27
Transgene copy number	1	∼3	∼7	∼10	∼25	∼35	∼65
Silencing frequency	<1×10^−7^	0.1±0.03%	0.002±0.001%	1±0.5%	90±9.5%	90±6.4%	95±4.8%

We also found that the high copy number of the transgene did not always lead to a high silencing frequency of the transgenic *ADE2* genes. For instance, we observed ∼10% red ade− colonies derived from PPW22 and PPW26 (Figure 1C and Figure S2). Only PPW27 exhibited a high silencing rate for the *ADE2* gene. In addition, quantitative real-time RT-PCR showed that the *ADE2* expression levels were much higher in PPW22 compared with those in the wild-type strain, while the levels of *CPA1* were virtually undetectable in PPW22, indicating that silencing of the endogenous *CPA1* gene and the transgenic *ADE2* gene might be two distinct events (Figure S3). In strains PPW25, *cpa1 cpa2*, and AAC1 (*gpa1::ADE2*) [27], which contain different copy numbers of the *ADE2*-based transgene, expression levels of the *ADE2* gene were also high and in accord with the numbers of the *ADE2* transgenes, suggesting lower silencing rates of the transgenic *ADE2* genes (Figure S3). Our results suggest that *ADE2* is less sensitive to silencing, and higher copy numbers or a specific transgene location may be required for its efficient silencing when compared with silencing of *CPA1/2*.

### Transgene silencing of the *CPA1* and *CPA2* genes involves RNAi

**Figure 4:**
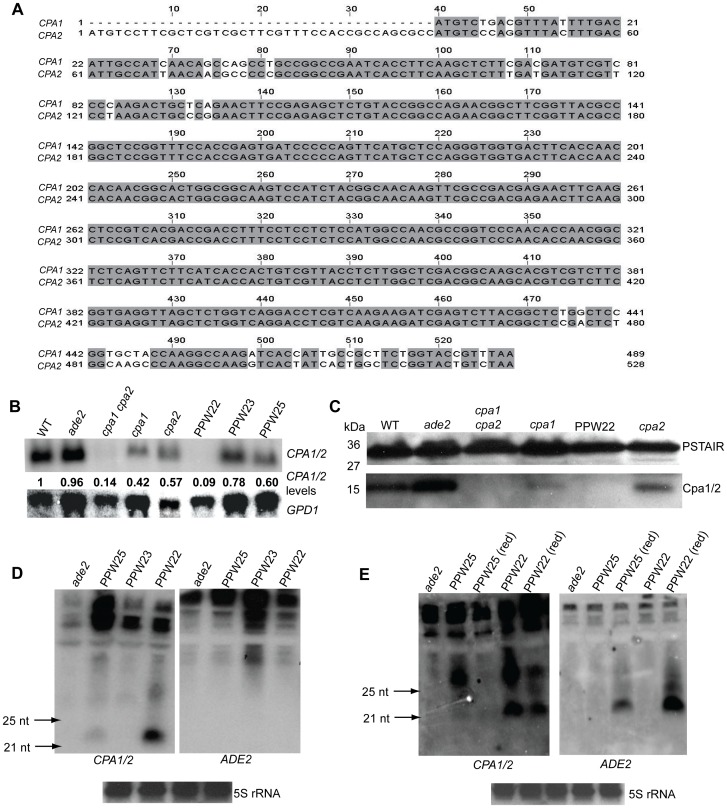
Silencing of the *CPA1* and *CPA2* genes in strains bearing a *cpa1::ADE2* transgene array. A) The *CPA1* and *CPA2* genes are highly conserved and share ∼93% nucleotide identity in the coding regions (introns are not depicted), including a region of 339 bp with 100% identity. Identical nucleotides are darkly shaded. B) Expression of *CPA1* and *CPA2* was examined by northern blot analysis. The full-length *CPA1* gene was used as a probe and hybridizes with both the *CPA1* and the *CPA2* mRNAs. The *GPD1* gene served as the loading control. The quantities of the *CPA1/2* gene mRNA were determined by phosphorimager analysis and Image Quantifier 5.2 software. The quantity of *CPA1/2* in each sample was compared with that in the wild-type strain (set as 1) after normalization with *GPD1*. C) Western blot detection of *C. neoformans* protein extracts indicates that the abundance of both the Cpa1 and Cpa2 proteins is reduced in the transgenic strain PPW22. Total proteins from the wild-type, *ade2*, *cpa1*, *cpa2*, *cpa1 cpa2*, and PPW22 strains were separated on a 4–20% SDS-PAGE gel, blotted, and incubated with anti-Cpa1 antiserum. The same membrane was stripped and probed with anti-PSTAIR antibodies to detect the Cdc2 protein as a loading control. D) *CPA1* siRNAs are present in the transformed strains exhibiting CsA resistance. Total RNAs were isolated and siRNAs were enriched and resolved in a 15% denaturing polyacrylamide TBE-urea gel and probed with a sense *CPA1* probe. Northern analysis revealed that *CPA1* siRNAs are highly abundant in transgenic strain PPW22, but much less abundant in PPW25. No siRNA was detected when the blot was probed with a ^32^P-labeled *ADE2* sense probe. E) Abundant *ADE2* siRNAs were only observed when RNA was extracted from red (ade^−^) colonies of the variegating strains (red/white). 5S rRNA was probed to serve as a loading control.

The observation that strain PPW22 was completely CsA resistant albeit encoding wild-type copies of the *CPA1* and *CPA2* genes indicates that introduction of the *cpa1::ADE2* transgene allele might result in silencing of both the *CPA1* and *CPA2* genes. To test this hypothesis, a northern blot was performed with a probe that hybridizes to both the *CPA1* and *CPA2* genes, which are highly conserved and share ∼93% overall nucleotide identity in the coding regions, including regions of 100% identity spanning two regions of 26 and 339 nucleotides (Figure 4A). Similar to the *cpa1 cpa2* double mutant strain, little or no *CPA1* or *CPA2* mRNA was detected in the PPW22 silenced strain. By contrast, much more *CPA1/2* mRNA accumulated in strains PPW23 and PPW25 with lower copy number transgene arrays (Figure 4B). Furthermore, western blotting using antiserum raised against Cpa1 and that cross-reacts with Cpa2 [25] documents that the Cpa1 and Cpa2 proteins are both considerably reduced in abundance in PPW22 (Figure 4C).

In the fungus *N. crassa*, multiple repeated transgenes often induce silencing through methylation, and the methylation inhibitor 5-azacytidine can increase the reversion frequency of some silenced strains [28]. Our observations suggest that a similar process does not operate in *C. neoformans*. First, growth of PPW22 or PPW25 cells on medium containing 5-azacytidine did not restore CsA sensitivity. Second, analysis of Southern blots on DNA digested with the methylation-dependent DNA endonuclease McrBC did not provide any evidence that methylated DNA was present (data not shown).

We then tested whether asexual co-suppression is dependent on the RNAi silencing pathway, similar to quelling in *N. crassa* that occurs during vegetative growth [29]. Because generation of siRNA is a hallmark of RNAi pathways, we examined the presence of siRNA by northern blotting. When probed with a ^32^P-labeled sense *CPA1* transcript, abundant siRNA of ∼22 nt were observed in PPW22 (25 transgenes), whereas only a very modest level was detected in PPW25 (3 transgenes), and none occurred in PPW23 (1 transgene) or the *ade2* strain M049 (Figure 4D). The observation of antisense siRNAs of *CPA1* suggests that siRNAs are derived from a dsRNA precursor and may function as a *trans*-acting factor to silence both *CPA1* and *CPA2*, by virtue of the high sequence identity *CPA2* shares with *CPA1*. In a duplicate blot probed with a ^32^P-labeled sense *ADE2* probe, no siRNA was detected specific for *ADE2*. Hybridization signals to *ADE2* siRNAs were only observed in samples of RNA extracted from red (ade−) colonies produced by variegation of strains PPW22 and PPW25 from white (Ade+) to red (ade−) (Figure 4E). Overall, these findings indicate that silencing of the endogenous *CPA1*/*CPA2* and transgenic *ADE2* genes both involve an RNAi pathway and the two silencing events appear to occur at different frequencies.

**Figure 5:**
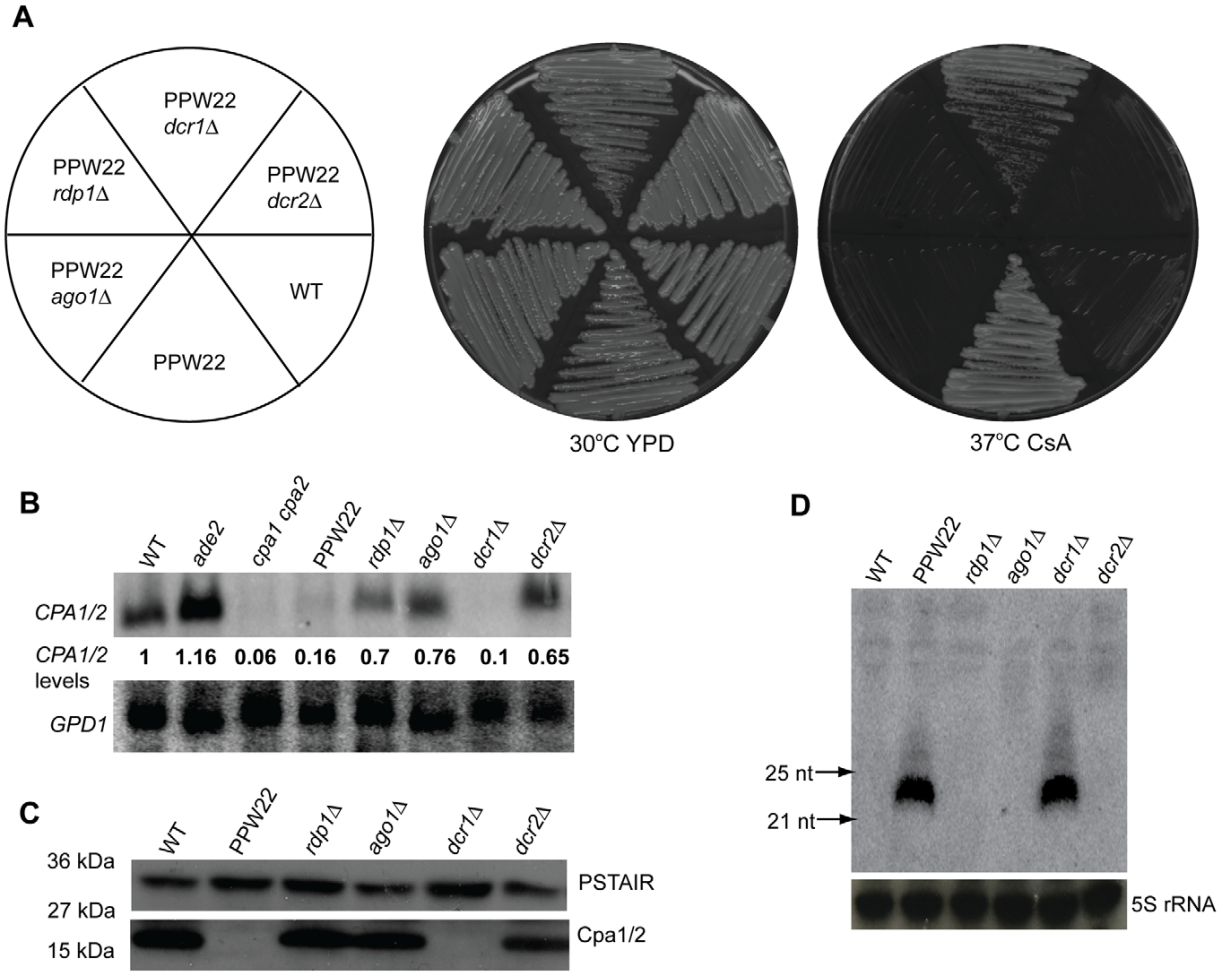
RNAi components are required for transgene-induced vegetative silencing. A) Transgenic strain PPW22 and *ago1Δ*, *rdp1Δ*, *dcr1Δ*, or *dcr2Δ* mutant derivatives were grown on YPD medium without or with 100 µg/ml CsA, incubated at 30°C and 37°C for four days, and photographed. B) Expression of *CPA1* and *CPA2* was examined by northern blot analysis. The quantities of *CPA1/2* were determined by phosphorimager analysis and Image Quantifier 5.2 software as described above. C) Expression of the Cpa1 and Cpa2 proteins was detected by immunoblotting with anti-Cpa1 antiserum. D) siRNAs were enriched (see Materials and Methods) from individual total RNAs and resolved in a 15% denaturing polyacrylamide TBE-urea gel. A ^32^P-labeled in vitro transcribed *CPA1* sense transcript was first hydrolyzed and then used to probe antisense *CPA1* siRNAs.

Previous studies have shown that the *C. neoformans* serotype A strain H99 genome encodes one Argonaute (Ago1), two Dicers (Dcr1 and Dcr2), and one RNA dependent RNA polymerase (Rdp1) [24], [30]. To further verify that the RNAi machinery is required for silencing, we deleted these components in the PPW22 strain. For each gene disruption, at least two independent deletion mutants were obtained and analyzed (Table S1 and Figure S4). As shown in Figure 5A, independently isolated *ago1Δ*, *rdp1Δ*, and *dcr2Δ* mutations completely reversed the CsA resistance phenotype to CsA sensitivity. Accordingly, the expression levels of the *CPA1*/*CPA2* genes and the Cpa1 and Cpa2 protein levels were restored to the WT levels in these RNAi mutant strains (Figure 5B and 5C). Additionally, *ago1Δ*, *rdp1Δ*, and *dcr2Δ* mutations blocked the generation of small RNAs corresponding to *CPA1/2* in PPW22 (Figure 5D). Deletion of *DCR1* had little or no influence on the CsA phenotype and the *dcr1Δ* mutant still contained a similar amount of siRNA as in the *DCR1* wild-type PPW22, suggesting that Dcr1 plays a minor role in the asexual co-suppression RNAi pathway compared with Dcr2. This is in agreement with our previous findings that Dcr2 plays the major role and Dcr1 a minor role in transgene-mediated sex-induced silencing [24].

### Replication Protein A, a ssDNA–binding protein complex, is essential for asexual co-suppression

Our finding that multiple copies of transgenes induce silencing of endogenous genes during vegetative growth of *C. neoformans* is similar to quelling in *N. crassa* [29]. The molecular mechanism of quelling has been extensively studied. It has been proposed that the production of transgene-specific aberrant RNA transcripts is the critical initial step in generating dsRNA precursors during an RNAi mediated pathway [29]. Recent studies have shown that an RNA-dependent RNA polymerase (RdRP) and a single-stranded DNA binding protein complex (RPA) play important roles in the production of aberrant RNA and the subsequent generation of dsRNA [31], [32]. RPA is a conserved eukaryotic heterotrimeric complex critical for DNA replication and repair [33], [34]. In mammals, it is composed of three subunits, Rpa70, Rpa32, and Rpa14, named according to their respective molecular masses [34]. The observation that 1) Rpa70 interacts with RdRP [32] and 2) the RPA complex is required for quelling in *N. crassa* [31] prompted us to test if RPA is involved in transgene-induced asexual co-suppression in *C. neoformans*.

**Figure 6:**
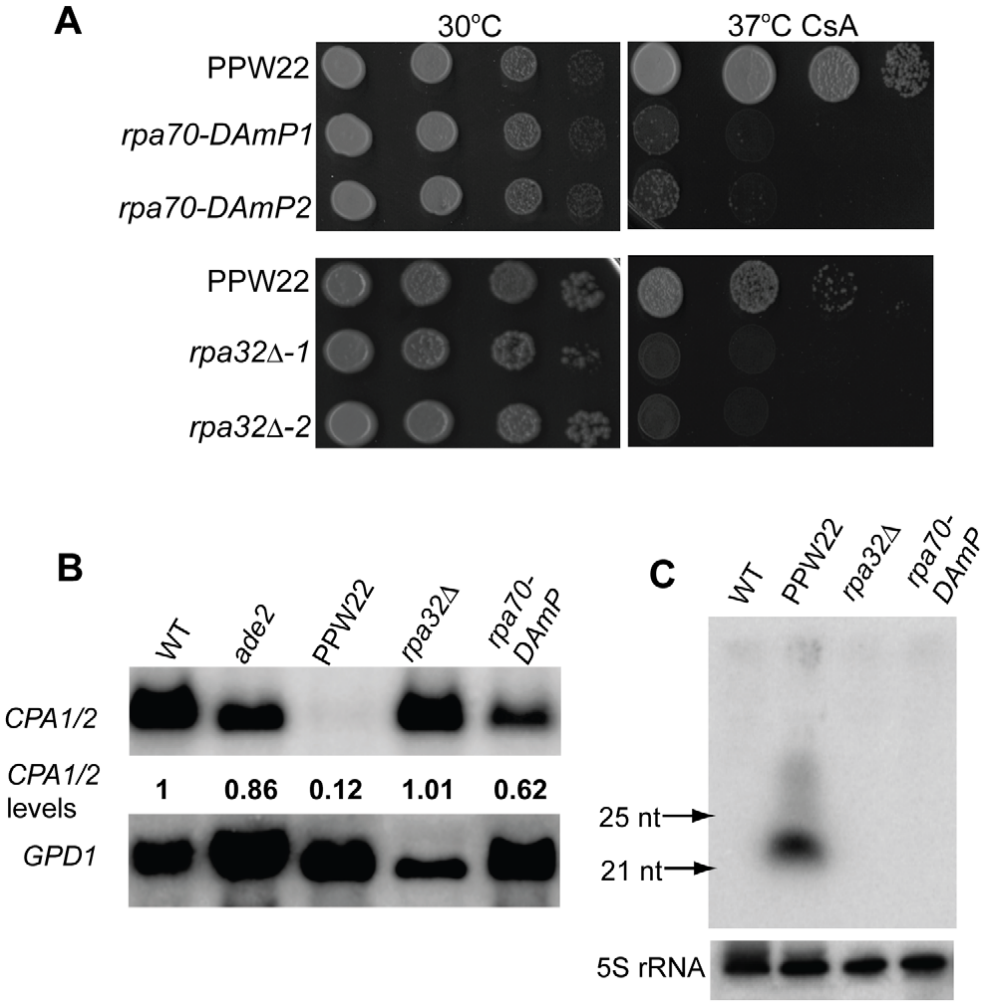
Replication Protein A, a ssDNA-binding protein complex, is essential for transgene-induced silencing. A) To examine the role of RPA subunits in asexual co-suppression, an *RPA70-DAmP* allele was constructed to reduce the amount of *RPA70* mRNA and the *RPA32* gene was deleted in transgenic strain PPW22. PPW22, two independent *rpa70-DAmP* mutant strains, and two independent *rpa32Δ* mutants were 10-fold serially diluted in YPD medium, and the cell suspensions were spotted onto YPD medium with or without CsA 100 µg/ml, incubated at 30°C and 37°C for four days, and photographed. B) Expression of *CPA1* and *CPA2* was examined by northern blot analysis. The abundance of *CPA1/2* were determined as described above. C) siRNAs were enriched from individual total RNAs and resolved in a 15% denaturing polyacrylamide TBE-urea gel. A ^32^P-labeled in vitro transcribed *CPA1* sense transcript was first hydrolyzed and then used to probe antisense *CPA1* siRNAs.

By analyses of the *Cryptococcus* genome database, we identified orthologs of *RPA70* (CNAG_01144.2) and *RPA32* (CNAG_01316.2) in *C. neoformans* as reciprocal best BLAST hits with the *N. crassa* RPA genes. We have been unable to identify an *RPA14* ortholog thus far. We first sought to generate *rpa* deletion mutations in the PPW22 transgenic strain to examine if the RPA complex functions in silencing. No progeny bearing an *rpa70Δ* mutant allele were obtained after sporulation of two independent *RPA70*/*rpa70Δ* diploid strains (Figure S5), and thus *RPA70* is essential (see Figure S6). We therefore constructed a “Decreased Abundance by mRNA Perturbation” (DAmP) allele of *rpa70* in strain PPW22 to reduce the mRNA expression levels of *RPA70*. An *rpa32Δ* haploid mutant was found to be viable. As shown in Figure 6A and 6B, independently isolated mutants bearing *rpa70-DAmP* or *rpa32Δ* mutations significantly reduced or abolished the CsA resistance silencing phenotype and restored the mRNA levels of *CPA1* and *CPA2*. In addition, siRNAs corresponding to the *CPA1/CPA2* genes were undetectable in the *rpa70-DAmP* and *rpa32Δ* mutants (Figure 6C). These results confirm that RPA is an important factor in the vegetative transgene silencing pathway. In addition, sexual development was defective during *rpa32Δ*×*rpa32Δ* bilateral mating: less hyphae formed, fewer basidia were produced, and no spore chains were observed (Figure S7). This sporulation defect makes it more difficult to assess whether RPA also plays a role during SIS.

## Discussion

We report the first example of a robust co-suppression pathway during *C. neoformans* vegetative growth and name it asexual co-suppression. This co-suppression shares characteristics with quelling identified in *N. crassa*, including gene specificity, a requirement for multiple transgene copies to trigger silencing, and varying degrees of gene silencing. Additionally, the fact that an RNAi pathway and the RPA complex are required for asexual co-suppression suggests that a common molecular mechanism operates in both asexual co-suppression and quelling. Our finding provides compelling evidence that RNAi serves to silence repetitive transgenes and transposons during vegetative growth in *C. neoformans*.

### RNAi silencing during vegetative growth of *C. neoformans*

The RNAi machinery is evolutionary conserved in a wide variety of fungal species [35]. Quelling, which was identified in *N. crassa* as the first RNAi silencing phenomenon reported in the fungal kingdom, is induced by transformation with transgenes that are homologous to an endogenous target [6], [7]. Even transgenes containing only fragments of genes led to inactivation of the corresponding native genes. Although RNAi silencing has been discovered in *C. neoformans* [30], [36], no similar robust transgene-induced silencing analogous to the canonical process of quelling had been reported. Hitherto, efficient and stable RNAi silencing during *C. neoformans* vegetative growth has always been achieved by introducing inducible constructs for dsRNA expression, including a hairpin RNA-expressing plasmid and an opposing-dual promoter system [36], [37]. We have previously reported an RNAi-related repeat silencing mechanism triggered by tandem multicopy transgenes in *C. neoformans*, whereas the silencing is most effective (∼50%) during sexual development and occurs at a very low frequency (∼0.1%) during vegetative growth [24].

SIS-RNAi silencing is important in defending the genome from transposons, as is quelling in *N. crassa*. Several lines of evidence support that RNAi silencing in *C. neoformans* also plays a role in transposon suppression during vegetative growth. First, our deep sequence siRNA library obtained from a mitotically growing strain reveals that abundant RNA sequences share homology with repetitive transposable elements [24]; second, a number of repetitive transposons are highly expressed or active in an *rdp1* mutant strain based on a comparative transcriptome analysis [24], [30]. However, the silencing rate during vegetative growth (0.1%) we observed previously is much lower than SIS (50%) when silencing *URA5* transgene arrays. Thus, the question here is whether there is an effective transgene-induced silencing during vegetative growth in *Cryptococcus*, like quelling in *N. crassa*. This study provides evidence that asexual co-suppression can be highly effective: transformed strains containing high copy numbers of the *cpa1::ADE2* transgene exhibited ∼90% silencing of the endogenous *CPA1* and *CPA2* genes. In this scenario, we hypothesize that RNAi may effectively suppress some highly repetitive transposons during vegetative growth, and SIS, a higher efficiency silencing mechanism deployed during the sexual cycle, prevents progeny from suffering assault by an even wider range of transposons and would otherwise occur when the two genomes of the mating parents are brought into contact with each other.

### Varying transgene silencing efficiency

We found that individual transformed strains bearing different copy numbers of *cpa1::ADE2* displayed variable efficiency of RNA interference: the silencing rate of the *CPA1* and *CPA2* genes reached ∼90% in the presence of 25 copies of the transgene while much lower or no silencing occurred in the transformants containing three- or a single-copy transgenes. The correlation between transgene copy number and silencing efficiency has also been observed in SIS [24]. However, in both cases, this correlation is not strict, as we have observed in strain PPW51 with multiple copies of transgenes a low silencing rate and SIS is saturated at a 50% with three or more copies of a transgene. We hypothesize that in addition to the copy number, the transgene arrangement and location may also contribute to the silencing frequency.

The presence of a high copy number of transgenes has been frequently found to be a prerequisite to trigger effective silencing. Two explanations have been proposed in plants. First, a high copy number of transgenes is necessary because transgenic mRNA accumulation needs to reach a threshold to induce specific degradation of both endogenous and transgenic mRNA [38]. However, evidence has been found in contradiction with this argument. For example, weakly or negligibly transcribed transgenes can efficiently induce PTGS [3] and high expression levels of a transgene are not sufficient to trigger gene silencing in *N. crassa* [7]. Thus, we favor an alternative hypothesis proposed by English *et al.* and Cogoni *et al.* [6], [39] that a qualitatively aberrant feature of transgenic DNA or RNA (aDNA or aRNA), rather than a high level of transgenic RNA accumulation, can trigger gene silencing. One plausible model is that RNA expressed from one repeat strand invades the DNA template of a neighboring repeat to form an aberrant RNA-DNA hybrid. This process could be facilitated by DNA/RNA helicases, RPA single strand DNA binding proteins, and both RdRP and DdRP activities. However, after silencing is initiated, the silencing efficiency is affected by additional factors, including growth conditions (vegetative vs. meiotic growth), and target gene expression levels (discussed more below).

We also noted that silencing of transgenic *ADE2* genes occurred at a much lower frequency compared with silencing of the endogenous *CPA1/2* genes in some transformed strains (PPW22 and PPW26), although both were introduced by the same transgene array. Robust silencing of both *CPA1/2* and *ADE2* was observed only in PPW27, with the presence of ∼65 copies of the *cpa1::ADE2* transgene at a different location. We hypothesize that *ADE2* is less sensitive to silencing, and inactivation of *ADE2* may require either higher copy numbers or a stricter location of the transgene. A similar case has also been observed when an interference plasmid incorporating portions of *CAP59* and *ADE2* between the opposing promoters was introduced to simultaneously silence both genes; however, colonies exhibiting only one mutant phenotype were found [36]. Based on these observations, we hypothesize that several factors may determine such variation in frequency. The first is that various mRNA expression levels between the target genes may dictate the silencing frequency. To observe a given mutant phenotype, for example CsA resistance compared to adenine auxotrophy, the mRNA must drop below a threshold that may differ for each target depending on its mRNA transcript levels and mechanism of action and regulation. This hypothesis can also explain our previous finding that silencing of transgenic *URA5* is extremely low during mitosis, but centromeric repetitive transposons that are barely expressed can be silenced by RNAi [24]. The other reasons for varying silencing efficiency could be the intrinsic characteristics of the genes determined by their base compositions, genome locations, and sequence context. The genes that are prone to form secondary structures could be more resistant to RNase activity, or the position of a gene locus within the nucleus may affect the efficiency of homology pairing.

### How is transgene silencing initiated?

A central question related to transgene silencing is: how do cells produce repetitive transgene-specific dsRNA to initiate RNAi? As discussed above, our observations support that it is not just the accumulation of transgenic RNA but rather the large tandem repetitive transgenic DNA itself that can be recognized and initiate aberrant RNA (aRNA) and dsRNA synthesis. With regard to quelling in *N. crassa*, dsRNA is generated by QDE-1 using the single strand repetitive DNA as the template because QDE-1 can act as both a DNA-dependent RNA polymerase (DdRP) and as an RNA-dependent RNA polymerase [31], [40]. More importantly, it is the RPA complex that recruits QDE-1 to the transgenic DNA repeats and promotes dsRNA formation [31]. Interestingly, we found that two components in the RPA complex, Rpa70 and Rpa32, are required for asexual co-suppression, suggesting that RPA may play a conserved role during transgene silencing in fungi. It will be of further interest to investigate how similar asexual co-suppression and quelling are at the molecular level, including examining whether Rdp1 in *C. neoformans* interacts with the RPA complex and whether it can function both as an RdRP and as a DdRP. Alternatively, a novel DdRP may remain to be identified that is responsible for the recognition of aberrant DNA repeats and to generate the initial dsRNA in *C. neoformans*.

## Materials and Methods

### Strains and media

The *C. neoformans MAT*α strain H99 has been previously described [41], [42]. M049 is an *ade2* strain derived from H99 following gamma irradiation, involving a chromosomal translocation event within the *ADE2* locus [41], [42]. All other strains used in this study are listed in Table S1. Yeast cells were grown and maintained on yeast extract-peptone-dextrose (YPD) media. Synthetic dextrose (SD) medium lacking adenine and YPD medium containing CsA (100 µg/ml) were used to test whether isolates of interest are auxotrophic for adenine or resistant to cyclosporine A. Mating of *C. neoformans* was conducted on 5% V8 juice agar medium (pH = 5) or Murashige and Skoog (MS) medium minus sucrose (Sigma-Aldrich), as previously reported [43].

### Gene disruption

A standard overlap PCR approach was used to disrupt genes of interest [44]. All mutations were generated in the serotype A background with the dominant *NAT* or *NEO* selectable markers. The overlap PCR products were introduced into the genome of recipient strains by biolistic transformation [45]. When constructing the DAmP mutant alleles, the *NEO* selectable marker was inserted immediately after the stop codon of the *RPA70* open reading frame by transformation with an overlap PCR product encoding the *NEO* marker flanked at each end with homology to the targeted locus [46], [47]. Primers that were used for amplification of the 5′ and 3′ flanking regions of each gene disruption cassette are listed in Table S2. Transformants were initially screened by PCR and Southern blot analyses were then conducted to identify a single integration at the desired locus.

### Real-time PCR

Quantitative real-time PCR assays were performed with primers specific to the actin gene *ACT1* and the *ADE2* and *CPA1* genes (Table S2) to determine the copy numbers of the *cpa1::ADE2* transgene. DNA of the wild-type strain H99 and transformed strains PPW22, PPW23, PPW25, PPW26, PPW27, PPW51, PPW52, and PPW75 that contain the *cpa1::ADE2* transgene were used as templates. PCR was conducted with Brilliant SYBR Green QPCR Master Mix (STRATAGENE) and the relative quantity of the *ADE2 or CPA1* gene determined by the ΔΔCt method according to the following equations: (Ct value means the threshold cycle) ΔCt = Ct (target) - Ct (normalize) and ΔΔCt = ΔCt (experimental) - ΔCt (control) Comparative expression level = 2^−ΔΔCt^.

### Chromosome separation using pulse field gel electrophoresis (PFGE)

The PFGE was carried out using a BioRad CHEF-DR II System. The plugs for the CHEF gel electrophoresis were prepared as previously described [42]. To separate chromosomal DNA, plugs were embedded in 0.5% agarose gel prepared using 0.5× TBE buffer as recommended in the user's manual, and the CHEF gel was run at 3 V/cm for 90 hours at 14°C, with 250 to 900 seconds ramp switch time. The gel was then stained using ethidium bromide and visualized under UV light.

### RNA extraction and northern blot analysis

Cells were grown in 5 ml of YPD medium overnight, harvested, washed, and lyophilized. Total RNA was extracted with TRIZOL Reagent (Invitrogen) following the manufacturer's instructions. For northern blot analyses, 10 µg of total RNA was separated by denaturing agarose gel electrophoresis and blotted to Hybond-N+ nylon membrane (Amersham). Genes of interest were PCR amplified with primers listed in Table S2, and labeled with [^32^P]-dCTP by the Rediprime II DNA labeling system (Amersham). The [^32^P]-dCTP-radiolabeled DNA fragments were hybridized to the membrane overnight and signals were detected with a Typhoon 9200 imager and Image Quantifier 5.2 software (Molecular Dynamics).

To detect siRNAs, total RNA from strains of interest was extracted with TRIZOL and RNAs of low molecular weight were first precipitated with 5% polyethylene glycol (MW8000) and 500 mM NaCl and separated in 15% TBE-Urea gels as previously described [4]. DNA oligonucleotides served as size markers. After electrophoresis, RNA was transferred to Hybond-N+ nylon membrane, and UV cross-linked. Single-stranded RNA probes were *in vitro* transcribed in the presence of ^32^P-labeled UTP with the MAXIscript T7 kit (Ambion), and hydrolyzed to smaller size (∼30–50 nt) by 80 mM sodium bicarbonate and 120 mM sodium carbonate at 60°C for 3 hours [4]. After hydrolysis, the RNA probes were added into the hybridization solution, and hybridized overnight at 42°C.

### Protein extraction and western analysis

Cells were grown in 50 ml YPD overnight and cell lysates were prepared as described [48]. The concentration of proteins was first established with Bio-Rad protein assay reagent and an equal amount (50 µg) of each protein sample was loaded into a 4–20% tris-glycine gel (Invitrogen). After gel electrophoresis, proteins were transferred to Immunoblot PVDF membrane (Bio-Rad), incubated with the polyclonal antisera against Cpa1 [25], and detected using the ECL system (Amersham Corp). Mouse monoclonal anti-PSTAIR antibody (abcam) against cyclin-dependent kinases served as a loading control. Anti-rabbit IgG horseradish peroxidase conjugate and anti-mouse IgG horseradish peroxidase conjugate (Amersham Corp) were used for secondary detection of membrane-bound rabbit and mouse primary antibodies.

### Determination of vegetative silencing frequency

To measure the vegetative silencing frequency in a given strain, 10 independent colonies were inoculated into 5 ml overnight cultures in YPD liquid medium. Cells were then harvested, washed, and plated on both YPD and YPD with 100 µg/ml CsA media at various dilutions. Colony numbers formed on CsA medium at 37°C and on YPD medium were counted from each individual culture and the silencing frequency was determined based on the colony forming units. The final reported silencing frequency is the mean of 10 individual silencing assays.

## Supporting Information

Figure S1 PPW26 and PPW27 exhibit different frequencies of *ADE2* silencing. PPW26 and PPW27 were inoculated into a 5 ml liquid overnight culture and then plated on YPD solid medium. The plate was photographed after 3 days growth at 25°C.
(TIF)

Figure S2 Transgenes in PPW22 are stably inherited during mitotic growth. PPW22 was grown 5 times on YPD medium and DNA was isolated from a colony on the first YPD plate (PPW22) and two single colonies on the 6^th^ plate (indicated as PPW22-new). DNA was also isolated from colonies that are sensitive to CsA (PPW22-CsA sensitive). Quantitative real-time PCR revealed the copy numbers of the transgene present in the genomes.
(TIF)

Figure S3 Silencing of the endogenous *CPA1* gene and the transgenic *ADE2* gene occurs at different efficiencies. Left: Quantitative real-time PCR revealed that different copy numbers of the *ADE2* based transgene are present in the genomes of the indicated transformed strains. Right: Quantitative real-time RT-PCR was employed to determine the expression levels of the *CPA1* and *ADE2* genes in the strains indicated.
(TIF)

Figure S4 Independently isolated *ago1Δ*, *rdp1Δ*, and *dcr2Δ* mutations reversed the CsA resistance phenotype to CsA sensitivity. A) Transgenic strain PPW22 and the second independent *ago1Δ*, *rdp1Δ*, *dcr1Δ*, or *dcr2Δ* mutant derivatives were grown on YPD medium without or with 100 µg/ml CsA, incubated at 30°C and 37°C for four days, and photographed. B) Expression of *CPA1* and *CPA2* was examined by northern blot analysis.
(TIF)

Figure S5 Southern blot analysis confirms the *rpa70* deletion in the diploid strain. Genomic DNA was prepared from the wild-type diploid strain (AI187) and eight transformed isolates (T1-4, T1-5, T1-7, T1-9, T2-13, T2-18, T2-19, and T2-20) that were previously identified as candidate deletion mutants by PCR. T1 and T2 indicate two independent biolistic transformation events. DNA was digested with XhoI and analyzed by Southern hybridization. Schematic restriction maps of the *RPA70* gene and the deletion allele are shown on the right. The blot was probed with a ^32^P-labeled *RPA70* fragment including the 5′ and 3′ UTRs. The arrows indicate the cleavage products from the deletion alleles. The *RPA70/rpa70Δ* heterozygous mutants used in this study are T1-4 (AI264) and T2-13 (AI265).
(TIF)

Figure S6 *RPA70* is essential in *C. neoformans*. The *RPA70/rpa70Δ* heterozygous mutants were generated by replacing one allele of the wild-type *RPA70* gene with a *NEO* selectable marker. Two independently isolated heterozygous mutants (AI264 and AI265) (see Table S1) bearing *rpa70Δ::NEO* were subjected to sporulation on MS medium. Basidiospores were collected and germinated on YPD medium. After germination, colonies were tested for growth on neomycin. No progeny (0/32) bearing an *rpa70Δ::NEO* mutant allele were obtained after sporulation of the *RPA70*/*rpa70Δ* diploid strains, indicating that the *RPA70* gene is essential. Colonies were also tested for growth on SD-adenine and SD-uracil, and mating type scored, indicating Mendelian segregation of these independent markers in the progeny. Plates show results of 23 progeny sporulated from strain AI265.
(TIF)

Figure S7 Sexual development is defective during *rpa32Δ*×*rpa32Δ* bilateral mating. Matings were performed on MS medium and mating hyphae and spores were photographed after 14 days of incubation in the dark at 25°C. Mating structures at 40× magnification (top), 100× magnification (middle), and 400× magnification (bottom). Bars: 20 µm (middle); 10 µm (bottom).
(TIF)

Table S1 Strains used in this study.
(DOC)

Table S2 Primers used in this study.
(DOC)
