# Supplementary figures and images for: Transgene Induced Co-Suppression during Vegetative Growth in Cryptococcus neoformans
Source: PLoS Genet. 2012 Aug 16;8(8):e1002885. doi: 10.1371/journal.pgen.1002885 (PMC3420925; doi:10.1371/journal.pgen.1002885)

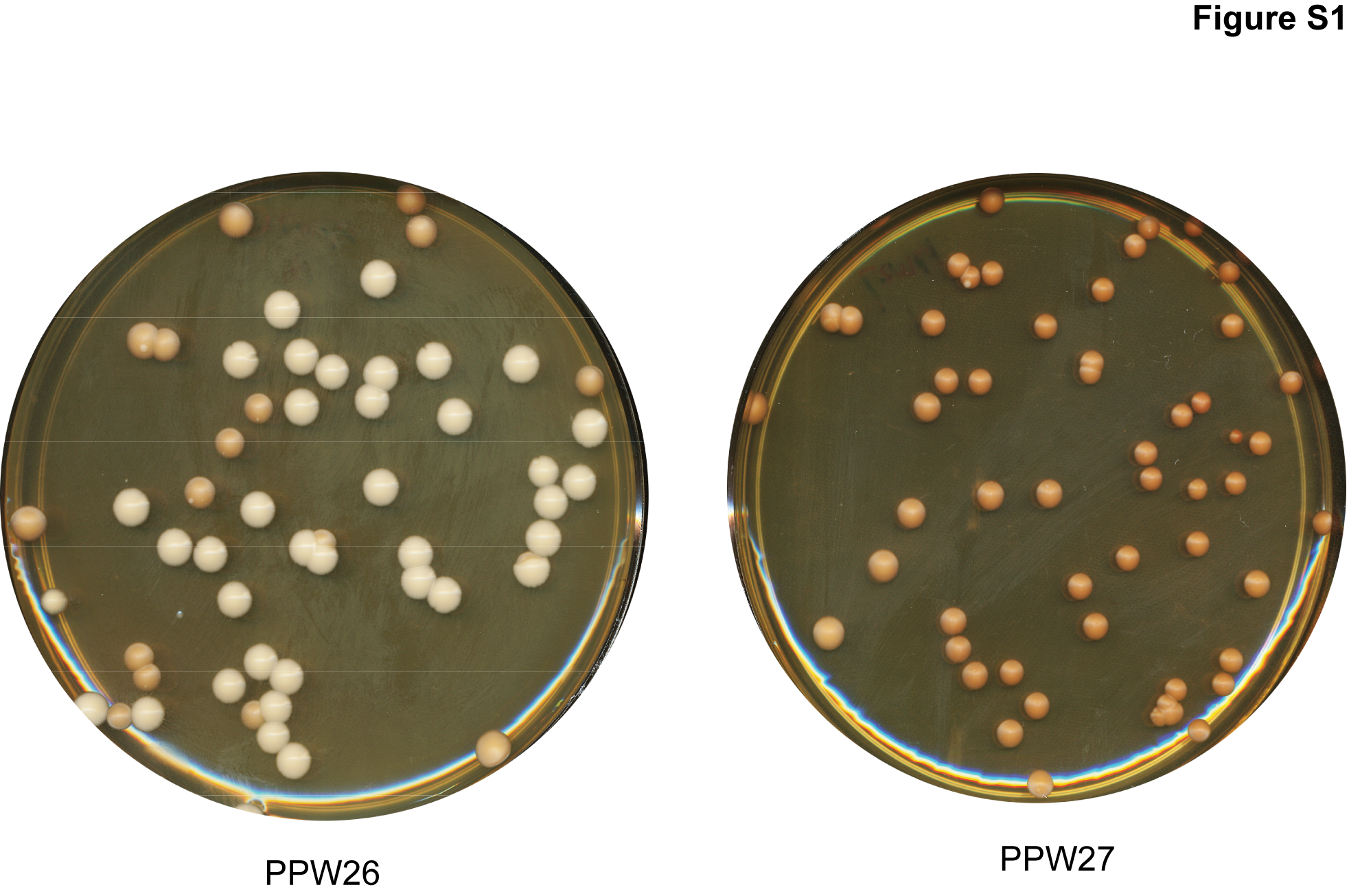

Supplement: Figure S1 — PPW26 and PPW27 exhibit different frequencies of ADE2 silencing. PPW26 and PPW27 were inoculated into a 5 ml liquid overnight culture and then plated on YPD solid medium. The plate was photographed after 3 days growth at 25°C. (TIF) [file pgen.1002885.s001.tif]

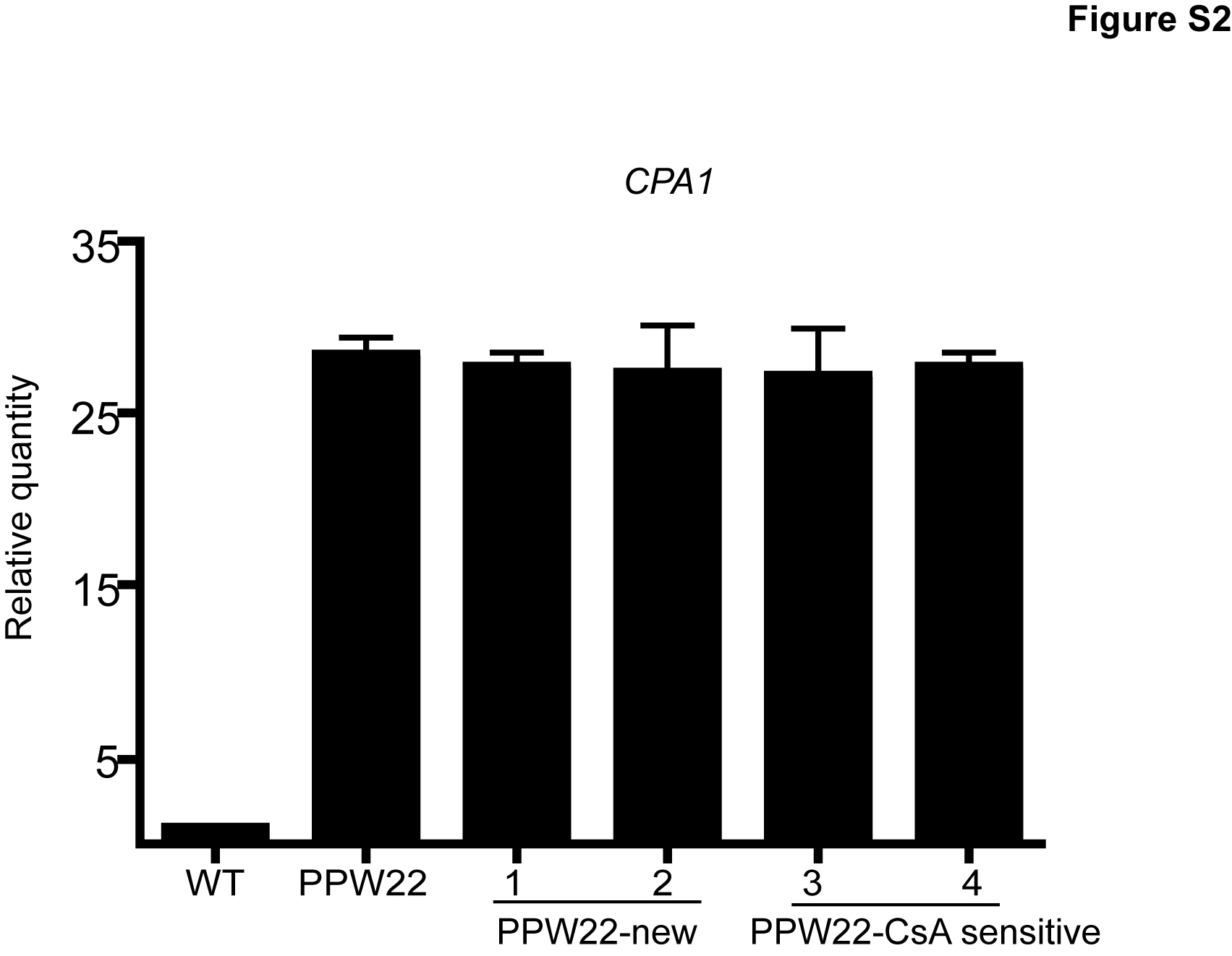

Supplement: Figure S2 — Transgenes in PPW22 are stably inherited during mitotic growth. PPW22 was grown 5 times on YPD medium and DNA was isolated from a colony on the first YPD plate (PPW22) and two single colonies on the 6th plate (indicated as PPW22-new). DNA was also isolated from colonies that are sensitive to CsA (PPW22-CsA sensitive). Quantitative real-time PCR revealed the copy numbers of the transgene present in the genomes. (TIF) [file pgen.1002885.s002.tif]

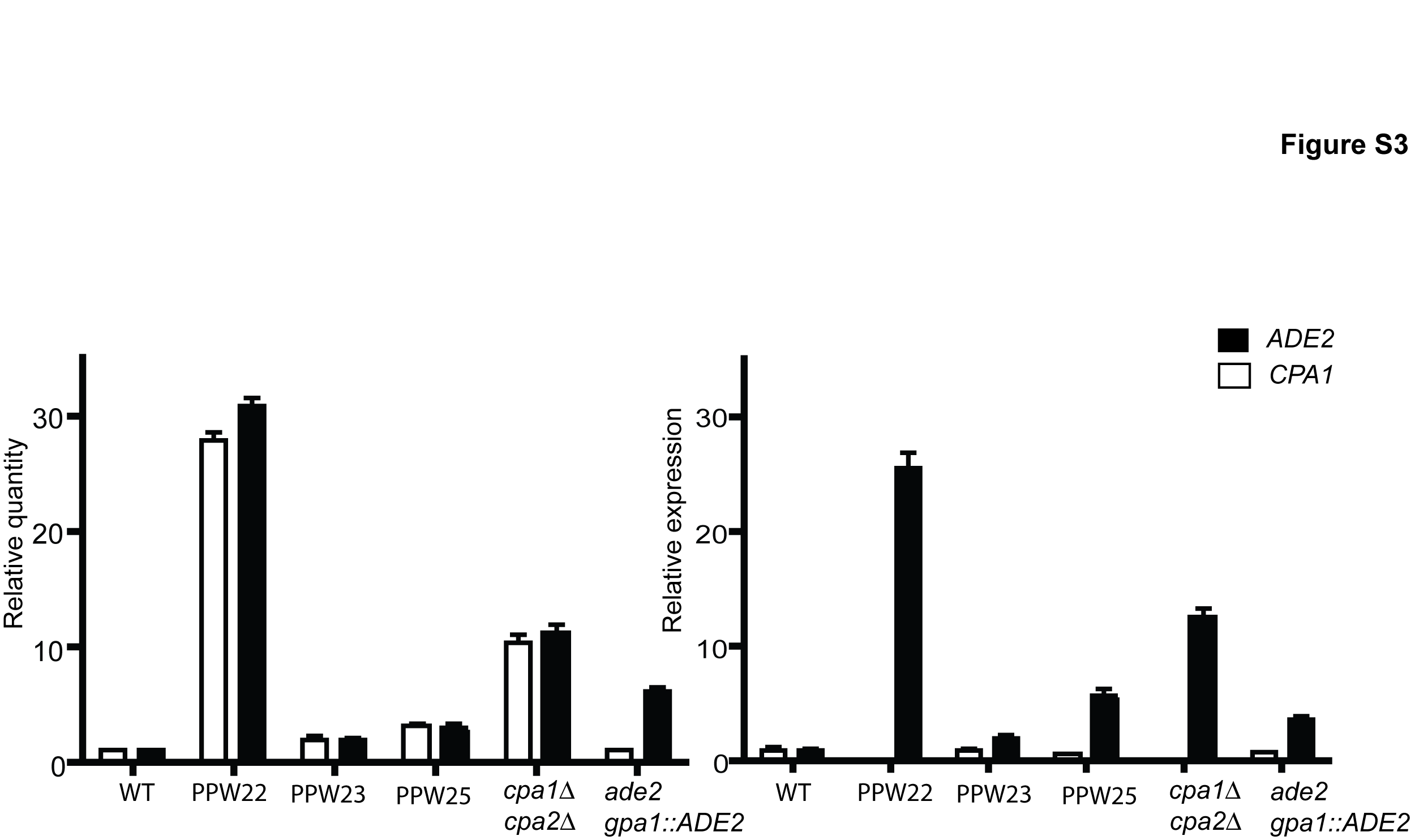

Supplement: Figure S3 — Silencing of the endogenous CPA1 gene and the transgenic ADE2 gene occurs at different efficiencies. Left: Quantitative real-time PCR revealed that different copy numbers of the ADE2 based transgene are present in the genomes of the indicated transformed strains. Right: Quantitative real-time RT-PCR was employed to determine the expression levels of the CPA1 and ADE2 genes in the strains indicated. (TIF) [file pgen.1002885.s003.tif]

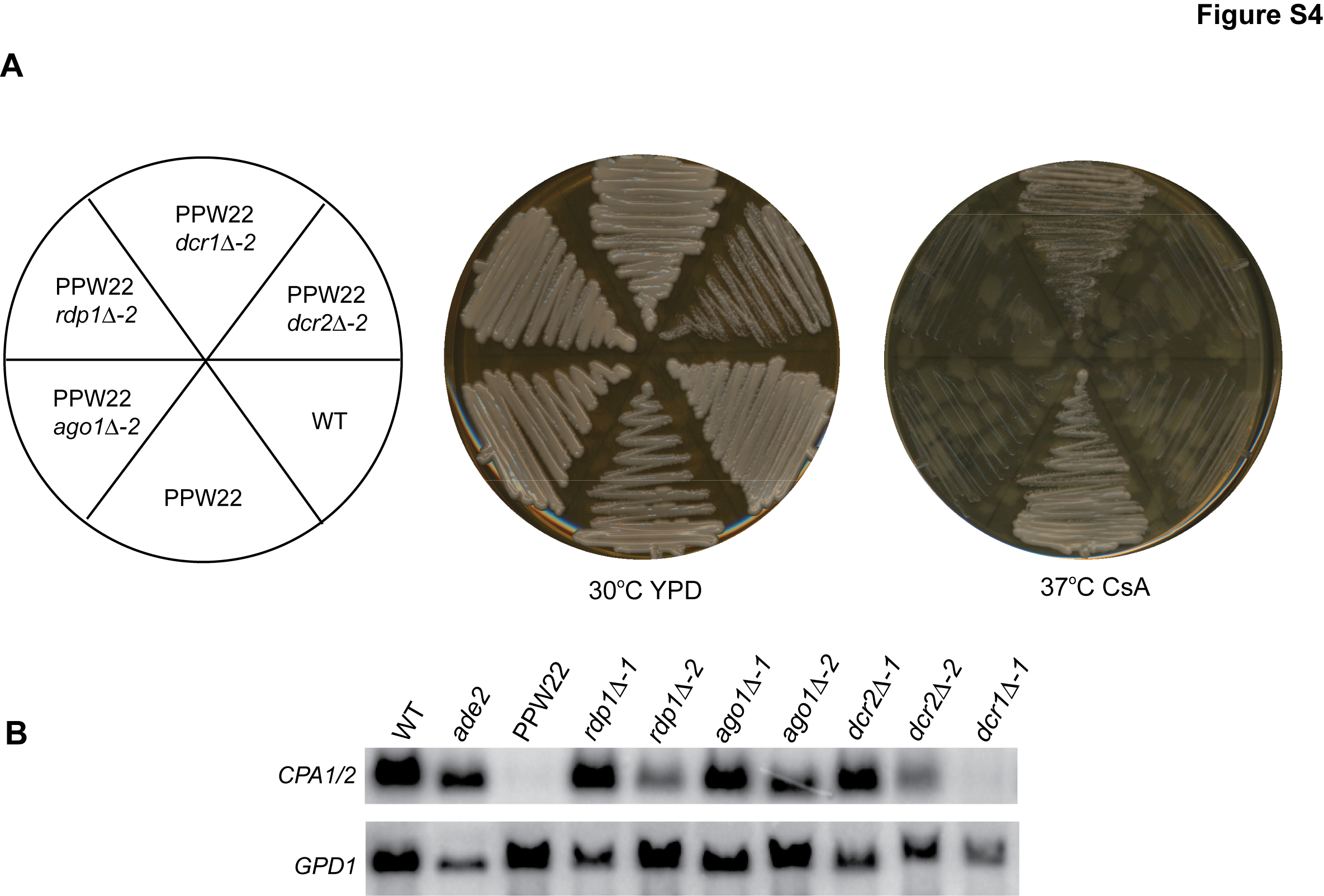

Supplement: Figure S4 — Independently isolated ago1Δ, rdp1Δ, and dcr2Δ mutations reversed the CsA resistance phenotype to CsA sensitivity. A) Transgenic strain PPW22 and the second independent ago1Δ, rdp1Δ, dcr1Δ, or dcr2Δ mutant derivatives were grown on YPD medium without or with 100 µg/ml CsA, incubated at 30°C and 37°C for four days, and photographed. B) Expression of CPA1 and CPA2 was examined by northern blot analysis. (TIF) [file pgen.1002885.s004.tif]

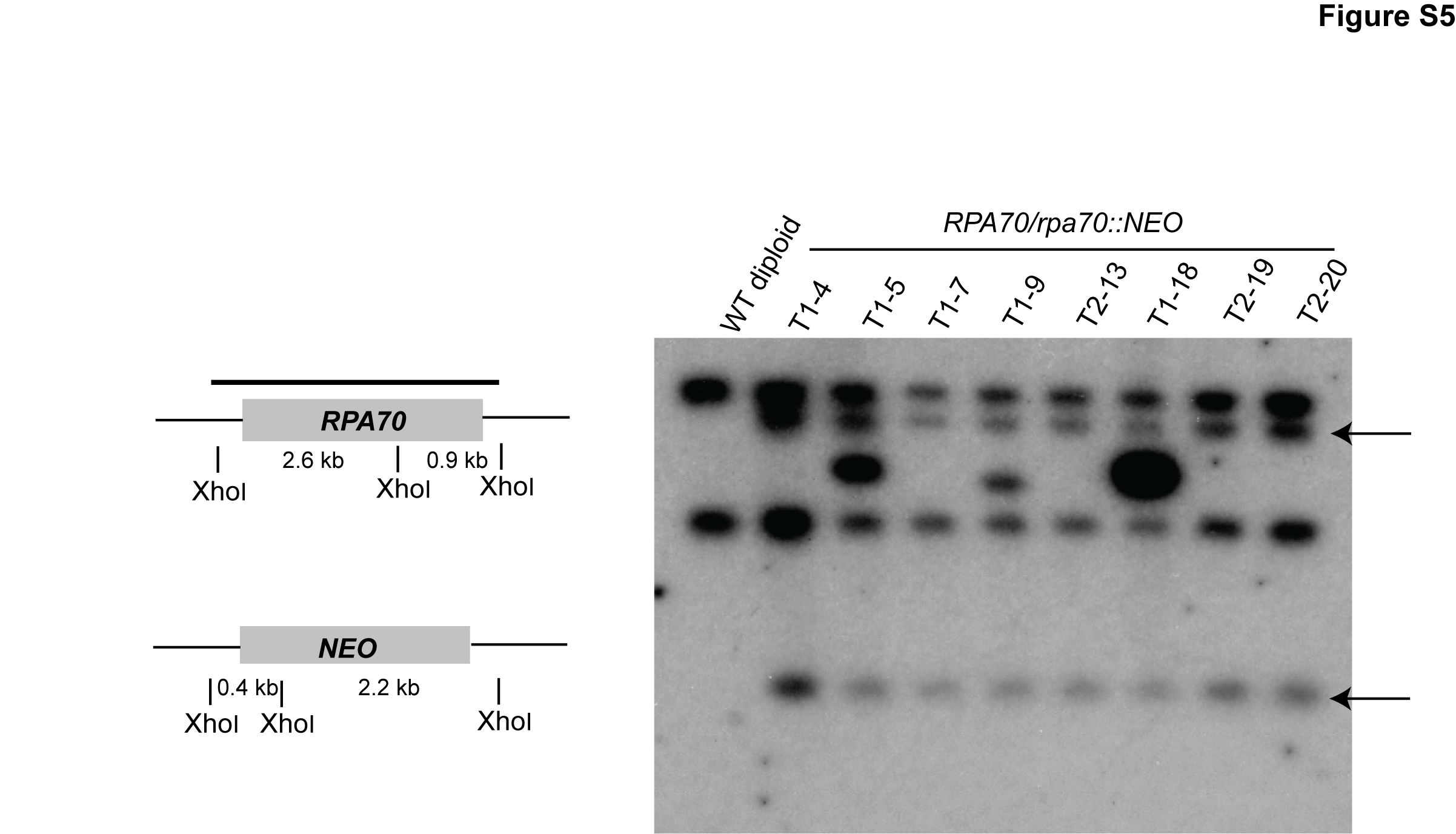

Supplement: Figure S5 — Southern blot analysis confirms the rpa70 deletion in the diploid strain. Genomic DNA was prepared from the wild-type diploid strain (AI187) and eight transformed isolates (T1-4, T1-5, T1-7, T1-9, T2-13, T2-18, T2-19, and T2-20) that were previously identified as candidate deletion mutants by PCR. T1 and T2 indicate two independent biolistic transformation events. DNA was digested with XhoI and analyzed by Southern hybridization. Schematic restriction maps of the RPA70 gene and the deletion allele are shown on the right. The blot was probed with a 32P-labeled RPA70 fragment including the 5′ and 3′ UTRs. The arrows indicate the cleavage products from the deletion alleles. The RPA70/rpa70Δ heterozygous mutants used in this study are T1-4 (AI264) and T2-13 (AI265). (TIF) [file pgen.1002885.s005.tif]

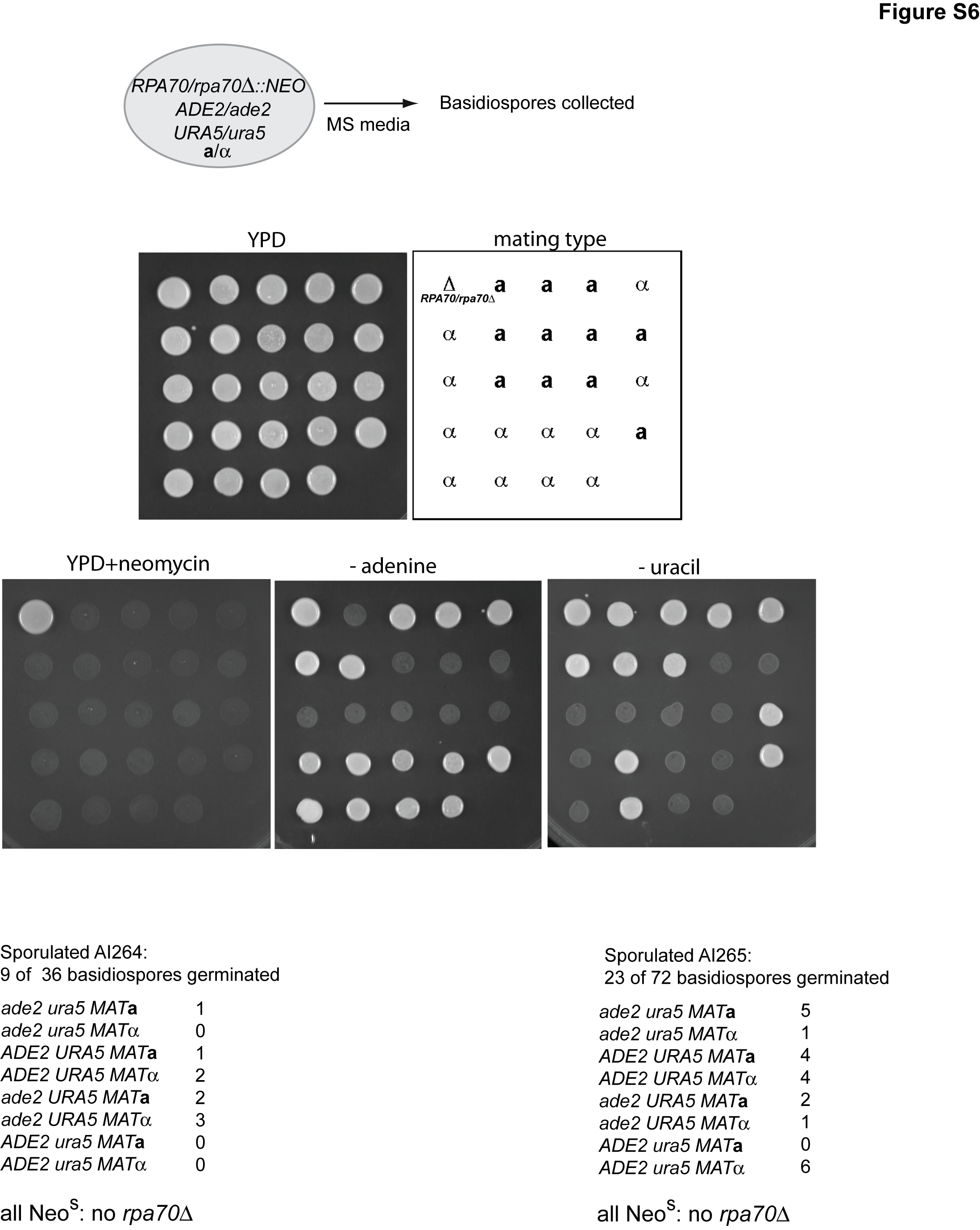

Supplement: Figure S6 — RPA70 is essential in C. neoformans. The RPA70/rpa70Δ heterozygous mutants were generated by replacing one allele of the wild-type RPA70 gene with a NEO selectable marker. Two independently isolated heterozygous mutants (AI264 and AI265) (see Table S1) bearing rpa70Δ::NEO were subjected to sporulation on MS medium. Basidiospores were collected and germinated on YPD medium. After germination, colonies were tested for growth on neomycin. No progeny (0/32) bearing an rpa70Δ::NEO mutant allele were obtained after sporulation of the RPA70/rpa70Δ diploid strains, indicating that the RPA70 gene is essential. Colonies were also tested for growth on SD-adenine and SD-uracil, and mating type scored, indicating Mendelian segregation of these independent markers in the progeny. Plates show results of 23 progeny sporulated from strain AI265. (TIF) [file pgen.1002885.s006.tif]

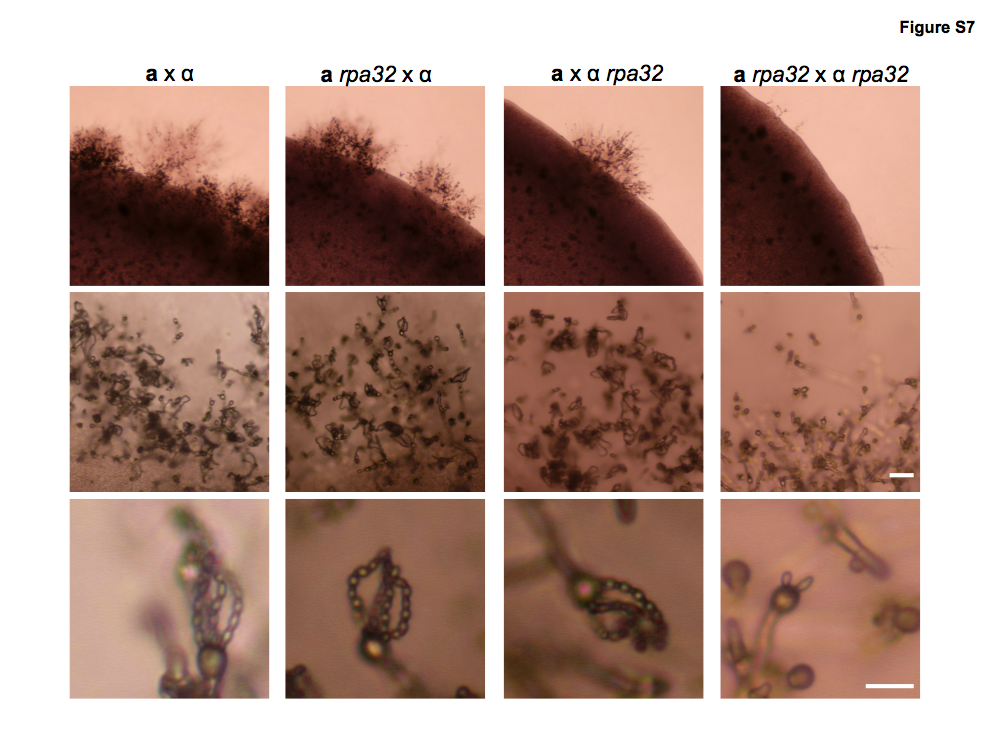

Supplement: Figure S7 — Sexual development is defective during rpa32Δ×rpa32Δ bilateral mating. Matings were performed on MS medium and mating hyphae and spores were photographed after 14 days of incubation in the dark at 25°C. Mating structures at 40× magnification (top), 100× magnification (middle), and 400× magnification (bottom). Bars: 20 µm (middle); 10 µm (bottom). (TIF) [file pgen.1002885.s007.tif]
