# Supplementary material for: Transgene Induced Co-Suppression during Vegetative Growth in Cryptococcus neoformans
Source: PLoS Genet. 2012 Aug 16;8(8):e1002885. doi: 10.1371/journal.pgen.1002885 (PMC3420925; doi:10.1371/journal.pgen.1002885)
Supplement: Table S1 — Strains used in this study. (DOC) [file pgen.1002885.s008.doc]

**Table S1. Strains used in this study.**

| Strain | Genotype | Parent | Source/Reference | |
| --- | --- | --- | --- | --- |
| *C. neoformans* var. *grubii* (serotype A, congenic to H99 and KN99**a**) | | |  |  |
| H99 | *MAT*α |  | [1] | |
| M049 | *MAT*α *ade2* | H99 | [1] | |
| F99 | *MAT*α *ura5* | H99 | [2] | |
| PPW22 | *MAT*α *CPA1 [cpa1::ADE2]_25_ ade2* | M049 | This study | |
| PPW23 | *MAT*α *CPA1 [cpa1::ADE2]_1_ ade2* | M049 | This study | |
| PPW25 | *MAT*α *CPA1 [cpa1::ADE2]_3_ ade2* | M049 | This study | |
| PPW26   |  |  |  |  |  | | --- | --- | --- | --- | --- | |  |  |  |  |  | | *MAT*α *CPA1 [cpa1::ADE2]_35_ ade2* | M049 | This study | |
| PPW27   |  |  |  |  |  | | --- | --- | --- | --- | --- | |  |  |  |  |  | | *MAT*α *CPA1 [cpa1::ADE2]_65_ ade2* | M049 | This study | |
| PPW51 | *MAT*α *[cpa1::ADE2]_7_ ade2* | M049 | This study | |
| PPW52 | *MAT*α *[cpa1::ADE2]_10_ ade2* | M049 | This study | |
| PPW60 | *MAT*α *cpa2::URA5 ura5* | F99 | [2] | |
| PPW75 | *MAT*α *cpa1::ADE2 ade2* | M049 | [2] | |
| PPW76 | *MAT*α *cpa1::ADE2 cpa2::URA5 ade2 ura5* | PPW60 | [2] | |
| XW81 | *MAT*α *CPA1 [cpa1::ADE2]_25_ ago1Δ::NAT* | PPW22 | This study | |
| XW82 | *MAT*α *CPA1 [cpa1::ADE2]_25_ ago1Δ::NAT* | PPW22 | This study | |
| XW83 | *MAT*α *CPA1 [cpa1::ADE2]_25_ rdp1Δ::NEO* | PPW22 | This study | |
| XW84 | *MAT*α *CPA1 [cpa1::ADE2]_25_ rdp1Δ::NEO* | PPW22 | This study | |
| XW85 | *MAT*α *CPA1 [cpa1::ADE2]_25_ rpa70-DAmP::NEO* | PPW22 | This study | |
| XW87 | *MAT*α *CPA1 [cpa1::ADE2]_25_ rpa70-DAmP::NEO* | PPW22 | This study | |
| XW159 | *MAT*α *CPA1 [cpa1::ADE2]_25_ rpa32Δ::NEO* | PPW22 | This study | |
| XW160 | *MAT*α *CPA1 [cpa1::ADE2]_25_ rpa32Δ::NEO* | PPW22 | This study | |
| SD1 | *MAT*α *CPA1 [cpa1::ADE2]_25_ dcr1Δ::NAT* | PPW22 | This study | |
| SD6 | *MAT*α *CPA1 [cpa1::ADE2]_25_ dcr1Δ::NAT* | PPW22 | This study | |
| SD2 | *MAT*α *CPA1 [cpa1::ADE2]_25_ dcr2Δ::NEO* | PPW22 | This study | |
| XW90 | *MAT*α *CPA1 [cpa1::ADE2]_25_ dcr2Δ::NEO* | PPW22 | This study | |
| XW158 | *MAT*α *rpa32Δ::NEO* | H99 | This study | |
| XW154 | *MAT***a** *rpa32Δ::NEO* | KN99**a** | This study | |
| AI187 | *MAT*α/**a** *ade2/ADE2 ura5/URA5* | JF99 and M001 | [3] | |
| AI264 | *MAT*α/**a** *ade2/ADE2 ura5/URA5 rpa70Δ::NEO* | AI187 | This study | |
| AI265 | *MAT*α/**a** *ade2/ADE2 ura5/URA5 rpa70Δ::NEO* | AI187 | This study | |
| AAC1 | *MAT*α *ade2 gpa1::ADE2* | M049 | [4] | |

indicates independently derived mutants.

The subscripted number after *[cpa1::ADE2]* indicates the transgene copy number. For example: *[cpa1::ADE2]_25_* means 25 copies of the transgene.

**References**

1. Perfect JR, Ketabchi N, Cox GM, Ingram CW, Beiser CL (1993) Karyotyping of *Cryptococcus neoformans* as an epidemiological tool. J Clin Microbiol 31: 3305-3309.

2. Wang P, Cardenas ME, Cox GM, Perfect JR, Heitman J (2001) Two cyclophilin A homologs with shared and distinct functions important for growth and virulence of *Cryptococcus neoformans*. EMBO Rep 2: 511-518.

3. Idnurm A (2010) A tetrad analysis of the basidiomycete fungus *Cryptococcus neoformans*. Genetics 185:153-63.

4. Sudarshan S, Davidson RC, Heitman J, Alspaugh JA (1999) Molecular analysis of the *Cryptococcus neoformans ADE2* gene, a selectable marker for transformation and gene disruption. Fungal Genet Biol 27: 36-48.
