# Supplementary material for: Transgene Induced Co-Suppression during Vegetative Growth in Cryptococcus neoformans
Source: PLoS Genet. 2012 Aug 16;8(8):e1002885. doi: 10.1371/journal.pgen.1002885 (PMC3420925; doi:10.1371/journal.pgen.1002885)
Supplement: Table S2 — Primers used in this study. (DOC) [file pgen.1002885.s009.doc]

**Table S2. Primers used in this study.**

| Primer | Sequence (5’ to 3’) | Comments |
| --- | --- | --- |
| JOHE8994 | TGTGGATGCTGGCGGAGGATA | universal screening oligo |
| JOHE17996 | TCGTAATCCGCAAGAGGTGC | *RDP1* deletion |
| JOHE17997 | CTGGCCGTCGTTTTACACCGCAGTGTGTTGTAAGGAATGTG | *RDP1* deletion |
| JOHE17998 | GTCATAGCTGTTTCCTGGGTGTTGTAAGGTCATAATCGG | *RDP1* deletion |
| JOHE17999 | GCGGAAGTTGGGTGGTACTGAG | *RDP1* deletion |
| JOHE18000 | TCGACAGGTTTAGTGTGTGTTGTT | *RDP1* screening |
| JOHE13547 | TTGGTGAGACTGGTCGTTGC | *AGO1* deletion |
| JOHE13548 | CTGGCCGTCGTTTTACGGTTGGTTTAGGTTTTCGC | *AGO1* deletion |
| JOHE13549 | GTCATAGCTGTTTCCTGGCGTCCTCTGTTATTCTTACGC | *AGO1* deletion |
| JOHE13550 | CATCATTCCTTGCTTTCCAC | *AGO1* deletion |
| JOHE18775 | GATGTTGCTCCCAAGGTTCT | *AGO1* screening |
| JOHE18776 | TAGCACTCTGTCGCATAGGA | *AGO1* screening |
| JOHE20877 | GCATTGTCTCCAACACATGG | *DCR1* deletion |
| JOHE20878 | CTGGCCGTCGTTTTACAGACACATTCTCCTCCCTCCA | *DCR1* deletion |
| JOHE20870 | GTCATAGCTGTTTCCTGGGTGTATATGGAATCGGTAT | *DCR1* deletion |
| JOHE20871 | GCGGGATGACCGGGCGTGAA | *DCR1* deletion |
| JOHE20928 | CTGACAGTCCCGTTCACCTTCAA | *DCR1* screening |
| JOHE20929 | ATTTCGAGGCGCTCATAGGTCCAT | *DCR1* screening |
| JOHE13540 | AGTTTCCGACTCTTGACGC | *DCR2* deletion |
| JOHE13541 | CTGGCCGTCGTTTTACCGGCTGATTGGAAAGTAAG | *DCR2* deletion |
| JOHE13542 | GTCATAGCTGTTTCCTGGTCTGAACTCAAGCGAGCCAC | *DCR2* deletion |
| JOHE13543 | TACAAGTCCCTCTTATCCCCCAGC | *DCR2* deletion |
| JOHE18777 | ATGACGGAGGAATCATCCTC | *DCR2* screening |
| JOHE18778 | GTCTGCTTGTTAACATGCA | *DCR2* screening |
| JOHE27009 | GGACCTCGTCAAGAAGATCG | *CPA1* QPCR |
| JOHE27010 | TCCTTTTCAATTCCCACGAG | *CPA1* QPCR |
| JOHE19493 | ATCGTTCTTGACTCTGGTGACGGT | *ACT1* QPCR |
| JOHE19494 | AAGTGGTGAAGAGGTAACCACGCT | *ACT1* QPCR |
| JOHE27146 | GATGTCCTTGAGGCTGTAGA | *ADE2* QPCR |
| JOHE27147 | AAGGCAAACCCAATCGACC | *ADE2* QPCR |
| JOHE26573 | GACATCTGTGCCCATGACAG | *CPA1* probe |
| JOHE26574 | ATTCCCACGAGCTCATCAAA | *CPA1* probe |
| JOHE26767 | CGATGAGCTCCACACACCC | *RPA32* deletion |
| JOHE26768 | CTGGCCGTCGTTTTACTGTTGCGGTGGAACTGGTTAT | *RPA32* deletion |
| JOHE26769 | GTCATAGCTGTTTCCTGGTTATGCAGTTCTGTTCGCAG | *RPA32* deletion |
| JOHE26770 | TCATTCAAAGCATTCTCCTGGG | *RPA32* deletion |
| JOHE27361 | GTCAACAACCGCAACAAATG | *RPA32* screening |
| JOHE27362 | GCTGCAAGATGAGATGGTCA | *RPA32* screening |
| JOHE26488 | CTGGCCGTCGTTTTACGACGAGGATGTCTCGCACCTCG | *RPA70* deletion |
| JOHE26489 | CATGGCTACATCTTCTAGGGC | *RPA70* deletion and DAmP |
| JOHE26490 | TCTTCTAGGGCAAACTCGCCC | *RPA70* screening |
| JOHE26491 | ACTTTCTTACTGCTCATATTGTCAG | *RPA70* deletion |
| JOHE26492 | GTCATAGCTGTTTCCTGCGGAATGATAAAGCAATGACAAATG | *RPA70* deletion |
| JOHE26493 | GCATTACTGCAAAGGAAATCGTAC | *RPA70* screening |
| JOHE26494 | GTCATAGCTGTTTCCTGTTATACGCTCATTTCGCTGATCAT | *RPA70* DAmP |
| JOHE26495 | GCAAGCTGAAACATTCCAAGCGGA | *RPA70* DAmP |
| JOHE26496 | CTGGCCGTCGTTTTACAAAAATTTCAGGGTAGCAGTCATCA | *RPA70* DAmP |
| JOHE22587 | TAATACGACTCACTATAGGGATGTCTGACGTTTATTTTGACATTG | T7 *CPA1* (in vitro transcription) |
| JOHE22588 | GTACCAGAAGCGGCAATGGTG | *CPA1* probe |
| JOHE22589 | ATGTCTGACGTTTATTTTGACATTG | *CPA1* probe |
| JOHE22590 | TAATACGACTCACTATAGGGGTACCAGAAGCGGCAATGGTG | T7 *CPA1* (in vitro transcription) |
| JOHE27225 | TAATACGACTCACTATAGGGATGGCACCCAGAAAGACGGTTGG | T7 *ADE2* (in vitro transcription) |
| JOHE27226 | GCATCTGACCAACATAGAACCG | *ADE2* probe |
| JOHE27227 | ATGGCACCCAGAAAGACGGTTGG | *ADE2* probe |
| JOHE27228 | TAATACGACTCACTATAGGGGCATCTGACCAACATAGAACCG | T7 *ADE2* (in vitro transcription) |
| JOHE27364 | TCAAGGTCGACGAACTACTTCCG | CNAG_01807 deletion |
| JOHE27365 | CTGGCCGTCGTTTTACGGATGATTCCAAGTATGCTTGG | CNAG_01807 deletion |
| JOHE27366 | GTCATAGCTGTTTCCTGAGAGTATCGCACCGAGCCAG | CNAG_01807 deletion |
| JOHE27367 | CCTTTGACTGCCTCTGGAAGACA | CNAG_01807 deletion |
| JOHE27368 | TGGAGAGAAAATCCCAATGC | CNAG_01807 screening |
| JOHE27369 | CATTTCTCCGAAGCTCCAAG | CNAG_01807 screening |
